# Interpretable QSAR and Complementary Docking for PARP1 Inhibitor Prioritization: Reliability Stratification and Near-Domain Screening

**DOI:** 10.3390/ph19040584

**Published:** 2026-04-07

**Authors:** Alaa M. Elsayad, Khaled A. Elsayad

**Affiliations:** 1 Biomedical Group, Department of Electrical Engineering, College of Engineering, Prince Sattam Bin Abdulaziz University, Wadi Alddawasir 11991, Saudi Arabia; 2 Pharmacy Department, Cairo University Hospitals, Cairo University, Cairo 11662, Egypt; khaled.al.elsayad@std.pharma.cu.edu.eg

**Keywords:** PARP1, QSAR, explainable AI, near-domain screening, molecular docking, lead prioritization

## Abstract

**Background/Objectives**: Poly(ADP-ribose) polymerase 1 (PARP1) is an important therapeutic target in DNA repair-deficient cancers, but discovery of new inhibitors remains constrained by scaffold convergence, tolerability limits, and acquired resistance. This study aimed to develop an interpretable, reliability-stratified cheminformatics workflow for PARP1 potency prioritization and structure-based follow-up. **Methods**: A curated ChEMBL dataset of 3339 PARP1 inhibitors was encoded using RDKit 2D descriptors and Avalon fingerprints (1143 initial features), then reduced to 132 informative variables by Random Forest-based feature selection. Five regression models were optimized, including a stacked ensemble. Model interpretation was performed using permutation feature importance and SHAP. External near-domain corroboration was assessed using a stringent PubChem similarity expansion (Tanimoto > 0.90) around sub-10 nM seed compounds, followed by comparison with retrievable experimental PARP1 activity values. Top scaffold-diverse candidates were further evaluated by complementary docking against PARP1 (PDB: 4R6E) using AutoDock Vina and cavity-guided docking through the SwissDock platform. **Results**: The stacked ensemble achieved the best held-out performance (test R^2^ = 0.723; RMSE = 0.610 pIC_50_ units), with 83.7% of test predictions within ≤0.75 pIC_50_ units and only 2.7% exceeding 1.5 pIC_50_ units. PubChem similarity expansion retrieved approximately 32,450 analogs, of which 3349 were predicted to have IC_50_ ≤ 10 nM. Among 366 compounds with retrievable experimental PARP1 activity values, predicted versus experimental pIC_50_ showed a positive association (R^2^ = 0.124; Pearson r = 0.479), with RMSE = 0.491 and MAE = 0.330 pIC_50_ units. Three ligands—CID 168873053, CID 175154210, and CID 172894737—showed the strongest complementary docking support and pocket-consistent poses relative to niraparib. **Conclusions**: This workflow provides a transparent and practically useful framework for near-domain PARP1 inhibitor prioritization. The combined QSAR, explainability, external corroboration, and docking strategy supports shortlist generation for experimental follow-up.

## 1. Introduction

Poly(ADP-ribose) polymerase 1 (PARP1) is a major sensor and effector of the DNA damage response. Upon binding DNA lesions, PARP1 catalyzes poly(ADP-ribosyl)ation (PARylation) of itself and nearby proteins, thereby coordinating chromatin remodeling, repair-factor recruitment, and repair-pathway choice across multiple DNA repair contexts. Because homologous recombination-deficient tumors rely heavily on compensatory repair programs, PARP inhibition can induce synthetic lethality, establishing PARP1 as a clinically important target in biomarker-defined cancers [1,2,3,4].

The clinical development of PARP inhibitors has reshaped treatment strategies in ovarian, breast, prostate, and pancreatic cancers. Their antitumor activity arises from two coupled mechanisms: catalytic inhibition of PARylation and trapping of PARP1 on damaged DNA, with the balance between these effects influencing both efficacy and toxicity. This mechanistic distinction has also stimulated interest in next-generation PARP1-selective inhibitors designed to preserve antitumor activity while reducing liabilities associated with broader PARP-family targeting [5,6].

Despite these advances, resistance to PARP inhibitors remains a major limitation. Reported mechanisms include restoration of homologous recombination, BRCA reversion events, replication-fork protection, altered drug transport, and PARP1-related adaptive changes [7]. In parallel, the recurrent use of closely related nicotinamide-mimetic chemotypes narrows accessible chemical space and may contribute to class-level vulnerability. Together, these challenges motivate the search for chemically differentiated PARP1 inhibitors with improved resistance resilience and clearer medicinal-chemistry decision support.

Data-driven ligand-based modeling offers a scalable route toward this goal. Recent QSAR- and AI-guided drug-discovery studies have highlighted the value of machine learning for extracting structure-activity relationships from large bioactivity datasets, while also emphasizing that interpretability is essential if predictions are to guide medicinal chemistry rather than serve as black-box rankings. In this context, explainable approaches such as permutation feature importance and SHAP can map predictive behavior onto chemically meaningful descriptors and fragment-level motifs, thereby improving model transparency and strengthening confidence in ML-guided molecular design [8,9].

Structure-based docking provides a complementary plausibility filter when interpreted conservatively. AutoDock Vina remains a widely used engine for rapid pose generation and relative ligand ranking, offering efficient sampling and scoring suitable for structure-based screening workflows [10]. In parallel, SwissDock 2024, within the SwissDrugDesign platform, integrates AutoDock Vina (ver. Vina Python library version 1.2.5.) with the enhanced Attracting Cavities 2.0 framework, enabling cavity-guided sampling and improved binding-site exploration within a unified web environment [11]. When used alongside ligand-based prioritization, these complementary docking analyses are best interpreted as qualitative evidence of pocket compatibility, geometric feasibility, and interaction recovery rather than as quantitative surrogates for experimental affinity.

The present study develops an interpretable PARP1 inhibitor discovery workflow that integrates curated bioactivity modeling, explainable AI, near-domain chemical-space expansion, and complementary docking analyses to prioritize structurally plausible candidates for experimental follow-up.

### Related Work

Poly(ADP-ribose) polymerase 1 (PARP1) is now recognized as a multifunctional genomic stress sensor that coordinates DNA damage recognition, PARylation signaling, replication-stress management, and chromatin remodeling, with additional roles in immune regulation and tumor microenvironment adaptation. These integrated functions shape both tumor evolution and therapeutic vulnerability. Recent reviews emphasize that although PARP1 inhibition has transformed the management of BRCA-mutated and homologous recombination-deficient (HRD) cancers, clinical benefit remains limited by predictable resistance mechanisms and dose-limiting toxicities, thereby motivating the development of next-generation inhibitors optimized for selectivity, resistance mitigation, and biomarker-guided patient stratification [12,13].

Mechanistically, PARP inhibitors (PARPi) exert antitumor activity through a dual mode of action comprising catalytic inhibition and PARP1 trapping. Agent-specific differences in trapping potency, chromatin engagement, and target residence time contribute to clinically meaningful heterogeneity. Contemporary analyses describe resistance as a multifactorial process involving restoration of homologous recombination, replication-fork protection, rewiring of DNA repair, drug-target decoupling, and activation of compensatory stress-response pathways. In this context, ATR/CHK1 signaling, which is activated during PARPi-induced replication stress, has emerged as a particularly relevant axis for overcoming both de novo and acquired resistance [14,15].

Disease context further modulates the translational value of PARP inhibition. In gliomas, where the biological rationale for PARP targeting remains strong, increasing support exists for rational combinations with radiotherapy, temozolomide, immunotherapeutics, anti-angiogenic agents, and targeted inhibitors. However, central nervous system-specific barriers—including limited blood–brain barrier permeability, intratumoral heterogeneity, and biomarker uncertainty—reduce the likelihood that biochemical potency alone will predict therapeutic success. Similarly, in BRCA-mutated, HRD, and triple-negative breast cancer (TNBC) settings, the field is increasingly moving toward combination strategies that intensify DNA damage or suppress adaptive survival pathways, particularly PI3K/AKT/mTOR signaling. At the same time, overlapping toxicities often compromise dose intensity, underscoring the need for discovery pipelines that prioritize candidates not only by potency, but also by mechanistic complementarity and potential combination compatibility [16,17].

In parallel, advances in computational chemistry have expanded the toolkit available for PARP1 inhibitor discovery. Recent surveys highlight the complementary value of QSAR modeling, docking, molecular dynamics, quantum-chemical calculations, and machine-learning workflows for accelerating hit identification and mechanism-consistent prioritization. Explainable AI frameworks, AutoML systems, and SHAP-based attribution approaches now support more transparent and generalizable small-molecule prediction pipelines. Structural bioinformatics studies further indicate that effective PARP1 virtual screening depends on recovery of canonical anchoring features, including polar interactions with Gly863/Ser904 and aromatic stacking with Tyr907/Phe897. Together, these findings support a hybrid validation strategy in which ligand-based predictions are cross-checked against conserved structural pharmacophores [18,19,20].

Collectively, the recent literature supports PARP1 discovery pipelines that are mechanism-aware, resistance-aware, and disease-context-aware, while remaining interpretable and externally corroborated. These requirements provide a clear rationale for integrated cheminformatics frameworks that combine predictive modeling, explainability, and orthogonal structure-based verification to enable more informed prioritization beyond potency alone.

## 2. Results

This section reports the outcomes of the QSAR modelling workflow, explainability analyses (PFI and SHAP), PubChem similarity–based external screening, and structure-based docking validation using AutoDock Vina. Collectively, these results quantify both the predictive strength and the mechanistic interpretability of the final stacked ensemble model.

A total of 3339 PARP1 inhibitors with exact IC_50_ measurements were retrieved from ChEMBL and converted to pIC_50_ values. Following molecular featurization using 1143 RDKit 2D descriptors and Avalon fingerprints (using RDKit extension version 5.2.0), the dataset was stratified into an 80% training subset (n = 2671) and a 20% held-out test subset (n = 668). Preprocessing—Z-score normalization, low-variance filtering, and correlation filtering—reduced the feature space to 1081 informative variables, reflecting substantial redundancy removal while preserving chemically meaningful variance. Subsequent supervised feature selection using RF importance produced a compact panel of 132 descriptors (~12% of the original feature space), yielding a parsimonious representation expected to mitigate overfitting while retaining key information on molecular size, aromaticity, functional-group composition, and fragment-level structural patterns relevant to PARP1 inhibition.

### 2.1. Model Optimization and Predictive Performance

Five regression models—random forest (RF), generalized linear model (GLM), gradient boosting (GB), artificial neural network (ANN), and a stacked ensemble—were optimized using random hyperparameter search coupled with 10-fold cross-validation. Mean Residual Deviance, which is numerically equivalent to mean squared error (MSE) under Gaussian regression, was used as the primary optimization criterion. Table 1 summarizes predictive performance on both the training set and the independent held-out test set.

On the training set, the GLM showed substantially weaker performance (R^2^ = 0.629) than the nonlinear models, consistent with the expectation that PARP1 inhibitor potency is governed by nonlinear structure–activity relationships that are not adequately captured by linear formulations. In contrast, RF, GB, and ANN all achieved strong training performance, indicating their ability to model complex descriptor–response relationships. The stacked ensemble performed comparably to the best individual learners on the training data (R^2^ = 0.946; RMSE = 0.277), as expected when integrating multiple high-performing base models.

On the independent held-out test set, the stacked ensemble achieved the best overall generalization (R^2^ = 0.723; RMSE = 0.610), outperforming all individual base learners. This result suggests that ensemble blending successfully leveraged complementary error profiles across RF, GB, ANN, and GLM, while descriptor preprocessing and RF-based feature selection reduced redundancy and noise without materially compromising the predictive signal. Overall, these findings support the robustness of the modeling pipeline and justify the selection of the stacked ensemble as the primary predictor for downstream virtual screening, explainable AI (XAI) interpretation, external corroboration, and docking-based structural assessment.

**Figure 1:**
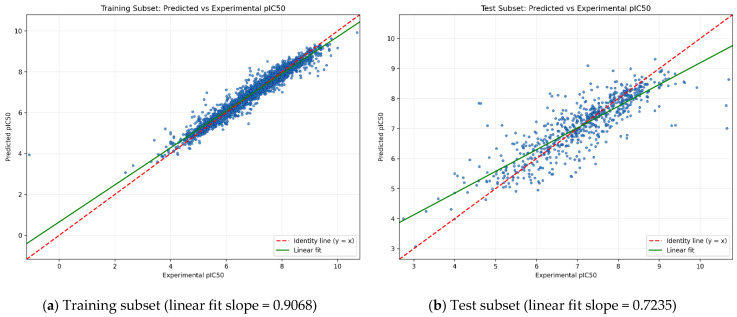
Predicted versus experimental pIC_50_ values for the stacked-ensemble model on the (**a**) training subset and the (**b**) held-out test subset. Each scatter plot displays the agreement between model predictions and experimental measurements. The dashed red line denotes the identity line (y = x), indicating perfect prediction, while the solid green line represents the fitted linear-regression trend, illustrating overall predictive bias and correlation strength [21].

Figure 1 shows predicted versus experimental potency for the stacked ensemble model, expressed as pIC_50_ (pIC_50_ = 9 − log_10_(IC_50_ [nM])), on the training (Figure 1a) and held-out test (Figure 1b) subsets. The observed-versus-predicted scatter plots show strong concordance between experimental and predicted pIC_50_ values, with the training data clustering closely around the identity line, consistent with high within-domain learnability. As expected under out-of-sample evaluation, dispersion increases in the test subset; nevertheless, the overall monotonic trend is preserved, supporting good generalization. The fitted regression slope was 0.9068 for the training subset and 0.7235 for the test subset, indicating mild compression of the predicted activity range in the held-out data. This behavior is typical of ensemble regression models and is often associated with limited representation of extreme potency regions. A small number of higher-residual points were also observed in the test set, consistent with the subsequent reliability analysis and underscoring the value of external corroboration and applicability-domain awareness for conservative screening decisions.

To further examine predictive reliability on the held-out test set, test predictions were stratified by absolute error into four regimes using a Failure Mode Atlas framework. Of the 668 test compounds, 306 (45.8%) had absolute errors ≤ 0.25 pIC_50_ units, and 253 (37.9%) fell between 0.25 and 0.75 pIC_50_ units, giving a cumulative 83.7% within ≤0.75 pIC_50_ units. A further 91 compounds (13.6%) fell between 0.75 and 1.5 pIC_50_ units, whereas only 18 compounds (2.7%) exceeded 1.5 pIC_50_ units. Thus, 97.3% of test predictions remained within ≤1.5 pIC_50_ units of the experimental value. The strong concentration of predictions in the low-error regimes indicates that the model is well suited for rank-based screening and prioritization, while the small failure subset motivates targeted error forensics and applicability-domain controls for conservative downstream triage.

### 2.2. Global Feature Importance: PFI and SHAP XAI

**Figure 2:**
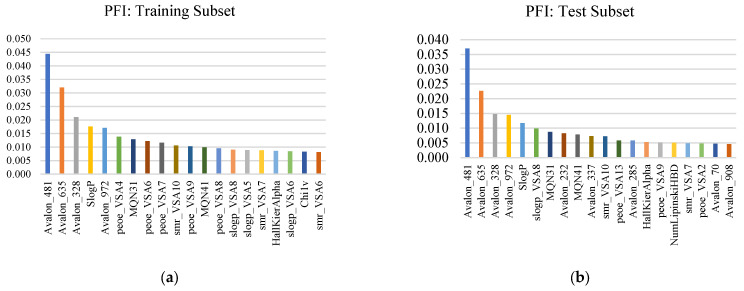
Top 20 permutation feature importance (PFI) descriptors for the (**a**) training and (**b**) held-out test subsets. PFI was computed as the decrease in explained variance (ΔR^2^) upon permuting each feature, with higher values indicating stronger global contribution to PARP1 pIC_50_ prediction.

Global interpretability was assessed using two complementary approaches—Permutation Feature Importance (PFI) and SHapley Additive exPlanations (SHAP)—to ensure that potency predictions are supported by stable, chemically meaningful drivers rather than split-specific artifacts. Figure 2 reports the top 20 PFI features for the training and test subsets, where importance is defined as the loss in explained variance (ΔR^2^) after permuting each feature. PFI revealed a highly consistent reliance structure across subsets: a small core of Avalon fingerprint bits (notably Avalon_481, Avalon_635, Avalon_328, and Avalon_972) dominated in both training and test data, indicating that predictive performance is primarily governed by reproducible fragment-level motifs characteristic of potent PARP1 chemotypes. Importantly, the persistence of the same top-ranked bits in the held-out test subset supports feature-level stability and argues against training-only signal. Beyond fragment drivers, secondary contributors were consistently drawn from global physicochemical and topology-derived descriptors, including SlogP, multiple VSA partitioning terms (e.g., PEOE_VSA, slogP_VSA8, smr_VSA), and scaffold complexity measures (e.g., MQN indices and HallKierAlpha), suggesting that potency is further constrained by lipophilicity and polar-surface/shape distributions. Comparison with the RF scaled-importance profile used for supervised filtering showed substantial agreement at the feature-family level, while modest rank reshuffling is expected given correlated predictors and the different definitions of importance (split gain vs. permutation performance loss). Together, these results support that the final 132-feature representation retains both fragment-driven SAR signal and the physicochemical envelope required for robust generalization.

**Figure 3:**
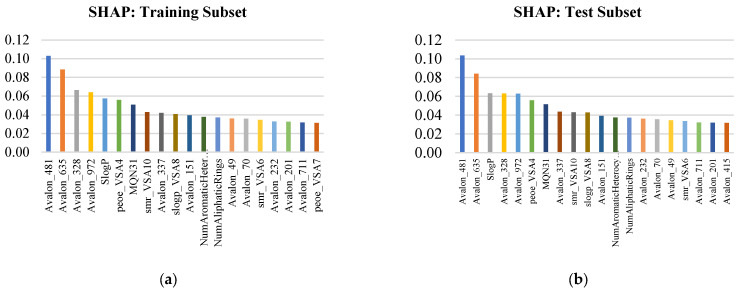
Top 20 global SHAP descriptors for the (**a**) training and (**b**) held-out test subsets, reported as mean absolute SHAP values. Higher values indicate features with larger average contributions to model predictions across compounds (importance magnitude, not effect direction).

Figure 3 summarizes global SHAP results for training and test subsets using mean absolute SHAP values, providing an attribution-based view of model dependence. SHAP likewise demonstrated strong stability under out-of-sample evaluation: 19 of the top 20 features were shared between training and test (Jaccard ≈ 0.91) with highly conserved ranking (Spearman ρ ≈ 0.98). As with PFI, SHAP identified the same dominant Avalon core (Avalon_481/635/328/972), reinforcing the conclusion that PARP1 potency prediction is anchored in consistent chemotype-level fragment signatures. Physicochemical descriptors (SlogP and VSA-family terms such as peoe_VSA4, smr_VSA10/smr_VSA6, slogP_VSA8) and complexity proxies (e.g., MQN31 and ring/heteroaromatic counts) contributed stable secondary influence, consistent with potency being modulated by lipophilicity–surface partitioning and scaffold topology. Overall, the convergence of PFI (ΔR^2^), SHAP, and RF importance provides a triangulated interpretability validation, indicating that the model’s predictive signal is chemically coherent, stable across splits, and suitable for hypothesis-driven SAR interpretation and downstream prioritization.

### 2.3. External Database Screening (PubChem) and Near-Domain Prospective Prioritization

To assess near-domain external generalization and enable prospective prioritization of PARP1 inhibitors, all ChEMBL compounds with reported IC_50_ < 10 nM were used as seeds for a stringent PubChem similarity expansion (Tanimoto > 0.90), yielding approximately 32,450 close analogs. Application of the finalized stacked-ensemble QSAR model to this similarity-expanded set prioritized 3349 compounds with predicted IC_50_ ≤ 10 nM, consistent with enrichment of potent candidates within chemical neighborhoods surrounding established high-affinity PARP1 chemotypes.

**Figure 4:**
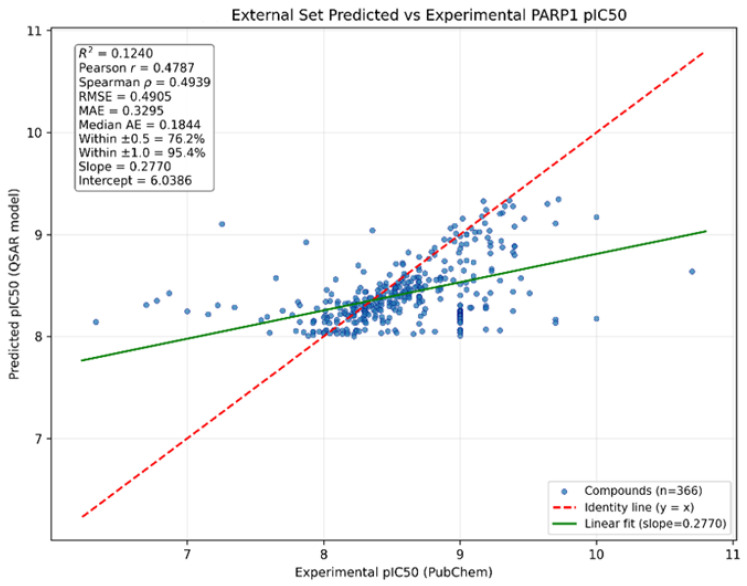
Predicted versus experimental pIC_50_ values for 366 PubChem similar inhibitors with retrievable PARP1 activity data. Both predicted and reported IC_50_ values were converted to pIC50 using pIC_50_ = 9 − log10(IC_50_ [nM]). The plot represents the external near-domain corroboration set used to assess model behavior prior to docking-based prioritization.

To further corroborate model behavior prior to docking-based triage, we examined similar inhibitors in PubChem for which experimental PARP1 inhibition data were available. A total of 366 compounds with retrievable PubChem PARP1 activity values were used for direct comparison between predicted and reported activities after conversion to pIC_50_ using pIC_50_ = 9 − log10(IC_50_ [nM]). As shown in Figure 4, the predicted and reported pIC_50_ values displayed a positive overall association, with Pearson r = 0.4787 and Spearman ρ = 0.4939, indicating that the model retained useful rank-ordering capability on this external near-domain set. In absolute-error terms, model performance was favorable, with RMSE = 0.4905 pIC_50_ units, MAE = 0.3295 pIC_50_ units, and a median absolute error of 0.1844 pIC_50_ units. Notably, 76.2% of compounds were predicted within ±0.5 pIC_50_ units of the experimental value, and 95.4% were within ±1.0 pIC_50_ units. Although the overall coefficient of determination was modest (R^2^ = 0.1240) and linear regression yielded a compressed slope of 0.2770, these results indicate that the model captures approximate potency levels and useful rank trends more effectively than the full experimental variance across the external set. Accordingly, the model appears well-suited for practical potency triage and shortlist generation in a near-domain screening context, supporting advancement of the top-ranked candidates to subsequent docking analysis.

The full set of 366 PubChem compounds used for the external comparison, including both predicted and experimentally reported PARP1 IC_50_ values, is provided in Supplementary Table S1.

All PubChem hits with predicted PARP1 IC_50_ ≤ 10 nM were retained for prospective structure-based follow-up only if they lacked both measured PARP1 IC_50_ values and prior PubChem PARP1 assay evidence. To preserve predicted potency while maximizing chemical diversity, the final docking shortlist was generated by ranking compounds by predicted IC_50_ and retaining the top Bemis–Murcko scaffold-diverse representatives [22]. This procedure yielded ten top-ranked, structurally distinct candidates for docking evaluation. These ten candidates were then advanced to an orthogonal consensus-docking plausibility layer against PARP1 using the crystal structure 4R6E. Docking was applied strictly as a structural compatibility filter, i.e., to test whether QSAR-prioritized ligands could adopt PARP1-consistent poses within the catalytic pocket rather than to provide an external measure of QSAR predictive accuracy. Both AutoDock Vina and Attracting Cavities (AC) 2.0 docking were performed through the SwissDock platform. Within this framework, AutoDock Vina was used for relative pose ranking and affinity-surrogate scoring (kcal/mol), whereas Attracting Cavities was used to improve robustness through cavity-guided sampling and complementary scoring.

All SwissDock calculations were performed using the same prepared receptor, with a docking box centered at (−91, 23, 43) and a box size of 20 × 20 × 20 Å. For the Attracting Cavities calculations, the protocol used medium sampling exhaustivity, 8 random initial conditions (RICs), and buried-cavity prioritization. SwissDock outputs were interpreted using the SwissParam-estimated ΔG (kcal/mol) as the primary energetic term for pose ranking, and the AC score as an auxiliary indicator of cavity compliance within the Attracting Cavities framework. The co-crystallized ligand 3JD (niraparib) from the PARP1–inhibitor complex (PDB: 4R6E) was included as an internal benchmark and pose-recovery reference under an identical receptor and site definition.

The final docking shortlist (Supplementary Table S2) was therefore selected to balance predicted potency, scaffold-level diversity, and prospective novelty. By applying Bemis–Murcko scaffold filtering after potency ranking, the procedure reduced the overrepresentation of closely related analog series and increased the likelihood of identifying structurally distinct ligands that engage the PARP1 pocket in different yet chemically plausible ways. Supplementary Table S3 summarizes the comparative AutoDock Vina results for Candidates 1–10 and the niraparib reference ligand. Because the SwissDock web server limits individual AutoDock Vina jobs to approximately 10 min, exhaustiveness could not be kept fully identical across all exploratory Vina runs; in each case, search depth was increased as much as permitted within the server runtime limit. Accordingly, Vina results were interpreted as provisional and supportive, whereas the fully standardized Attracting Cavities results were used as the primary basis for final docking prioritization. Supplementary Table S4 presents the comparative Attracting Cavities results for Candidates 1–10 and the niraparib reference ligand. In contrast to the exploratory Vina runs, the Attracting Cavities calculations were performed under fully standardized settings across all ligands, allowing direct comparison of cavity-guided docking scores and providing the principal basis for the final consensus ranking.

Table 2 shows the final consensus docking summary for Candidates 1–10 and the niraparib reference ligand. This table integrates predicted IC_50_, AutoDock Vina score, Attracting Cavities (AC) SP-dG, main pocket residues, and the final selection decision. Final ranking was weighted more heavily toward AC SP-dG, because the AC calculations were performed under fully standardized conditions across all ligands, whereas the exploratory Vina runs used non-uniform exhaustiveness. Accordingly, Vina was used as a complementary score and pose-plausibility check.

**Figure 5:**
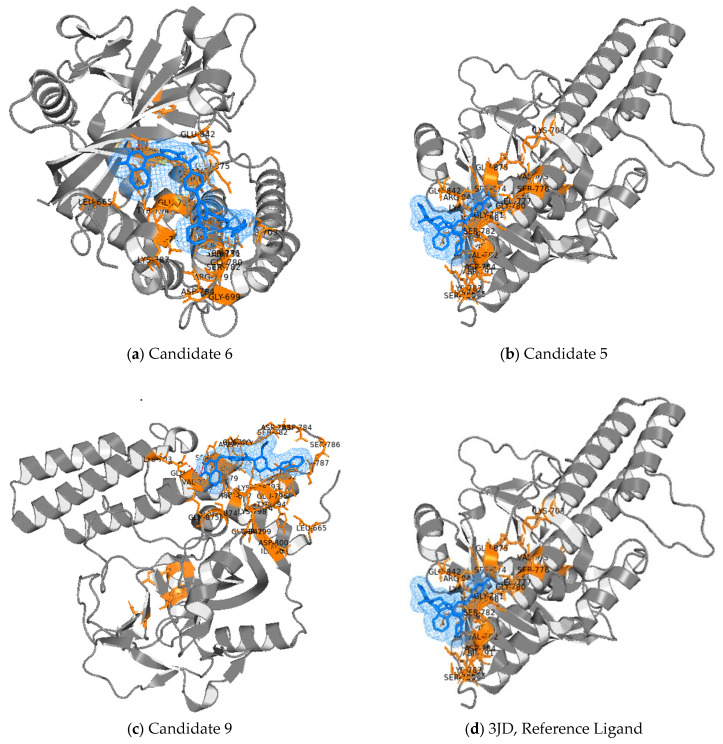
Pocket-consistent pose recovery for QSAR-prioritized PARP1 ligands using AutoDock Vina. Representative top-ranked AutoDock Vina poses of the three selected QSAR-prioritized ligands (Candidates 6, 5, and 9) are shown in the PARP1 catalytic pocket of 4R6E and compared with the co-crystallized reference inhibitor 3JD (niraparib; CID 24958200) under an identical receptor and search-space definition. PARP1 is shown as a gray cartoon, nearby binding-pocket residues as orange sticks, and docked ligands as blue sticks. The recovered poses support pocket-consistent accommodation of the selected ligands and provide a structural basis for their progression from QSAR prioritization to docking-supported candidate selection. Top-scoring poses were inspected using PyMOL (ver. 4.6.0) [23].

Based on the combined docking evidence, Candidates 5, 6, and 9 were selected as the top three ligands because they showed the most favorable Attracting Cavities SP-dG values, together with supportive AutoDock Vina scores and plausible localization within the PARP1 pocket. Candidate 3 was retained as a high-interest reserve compound because it yielded the most favorable Vina score overall while still showing supportive cavity-guided docking. As shown in Figure 5, the three selected ligands recovered plausible pocket-localized poses within the PARP1 catalytic region and displayed binding-site engagement broadly consistent with the reference niraparib orientation.

### 2.4. Structural Interpretation of Docking Results for the Top Three Predicted Inhibitors

To translate the consensus docking results into a medicinal chemistry context, the three highest-priority ligands were examined with respect to scaffold architecture, anchoring logic, and implications for developability. The selected compounds—CID 168873053 (Candidate 6), CID 175154210 (Candidate 5), and CID 172894737 (Candidate 9)—all share a phthalazinone core, but differ substantially in peripheral heterocyclic decoration, polarity distribution, and likely optimization trajectory. This combination of a conserved PARP1-compatible anchor with a diversified substituent space is favorable for lead discovery because it preserves mechanistic coherence while expanding options for follow-up optimization. Table 3 provides a concise SAR-oriented comparison of the three top-ranked docking-supported hits relative to the crystallographic reference niraparib, highlighting differences in scaffold profile, medicinal-chemistry interpretation, and recommended follow-up.

#### 2.4.1. PubChem CID 168873053 (Candidate 6)—Multi-Pharmacophore Phthalazinone with Solubilizing Motifs

N-[4-[4-[4-[2-fluoro-5-[(4-oxo-3H-phthalazin-1-yl)methyl]benzoyl]piperazin-1-yl]-6-morpholin-4-yl-1,3,5-triazin-2-yl]phenyl]-4-methylpiperazine-1-carboxamide

Candidate 6 emerged as the top consensus docking hit, combining a favorable AutoDock Vina score (−7.486 kcal/mol) with the most favorable Attracting Cavities SP-dG value (−8.5027 kcal/mol) among the ten shortlisted ligands (Table 2). Its top-ranked poses consistently localized within a PARP1-compatible pocket region and were associated with recurrent contacts involving LEU778, ARG779, VAL679, ILE790, ASP784, GLU795, LYS796, and LYS798, supporting a stable and chemically plausible binding pattern within the catalytic cleft.

Structurally, CID 168873053 is a highly functionalized phthalazinone bearing piperazine-, morpholine-, and triazine-associated heteroatom motifs, giving it the most polarity-rich profile among the three selected ligands. This architecture is consistent with a medicinal-chemistry strategy in which potency is pursued together with physicochemical tractability, an important consideration in phthalazinone-derived PARP1 series, where polarity tuning often determines whether enzyme-level potency can translate into useful exposure and downstream developability [24]. Taken together, its favorable consensus docking profile and solubilizing structural features identify CID 168873053 as the most lead-like candidate in the set. It should therefore be prioritized for biochemical PARP1 confirmation, together with PARP-trapping or residence-time-sensitive assays and early solubility and metabolic-stability profiling [25].

#### 2.4.2. PubChem CID 175154210 (Candidate 5)—Patent-Contextualized Dual-Target Hypothesis (PARP1–PI3K)

4-[[3-[4-[6-(6-amino-3-pyridinyl)-2-morpholin-4-ylpyrimidin-4-yl]piperazine-1-carbonyl]-4-fluorophenyl]methyl]-2H-phthalazin-1-one

Candidate 5 ranked second in the consensus structure-based evaluation, combining a favorable AutoDock Vina score (−7.356 kcal/mol) with a strong Attracting Cavities SP-dG value (−8.0426 kcal/mol) (Table 2). Its top-ranked poses consistently recovered a coherent interaction pattern across the PARP1 catalytic region, notably involving LEU777/LEU778, ARG779, ASP783/ASP784, TYR794, GLU795, LYS796, and LYS798, supporting stable accommodation within a PARP1 inhibitor-compatible binding environment.

From a medicinal-chemistry perspective, CID 175154210 is distinguished by a phthalazinone anchoring core linked to nitrogen-rich, kinase-like heterocyclic motifs, giving rise to a hybrid architecture compatible with PARP1–PI3K dual-target design logic reported in the patent literature. For example, WO2019100743A1 describes benzofuran-based PARP1–PI3K dual inhibitors, whereas US20210179610A1 discloses pyridopyrimidine-derived chemotypes designed to co-inhibit PARP and PI3K-family kinases [26,27]. Although the present study does not claim patent-aware filtering or direct dual-target activity, these precedents provide a useful contextual framework for interpreting the scaffold.

This hypothesis is also mechanistically relevant because PI3K/AKT signaling is a recognized adaptive survival pathway in acquired resistance to PARP inhibition, and suppression of this axis has been proposed as a strategy to restore PARPi sensitivity in resistant tumor settings [28]. Within this context, Candidate 5 appears less explicitly solubility-engineered than Candidate 6, but more strongly aligned with resistance-aware, mechanism-oriented follow-up. Taken together, its favorable docking profile, pocket-consistent interaction pattern, and scaffold-level alignment with published PARP1–PI3K design logic identify CID 175154210 as the strongest candidate for mechanism-informed follow-up. Accordingly, it should be advanced to biochemical PARP1 validation, orthogonal PI3K enzymatic profiling, and early ADME/PK triage to assess its potential as a dual-mechanism lead.

#### 2.4.3. PubChem CID 172894737 (Candidate 9)—Balanced Reserve Chemotype for Scaffold Diversification

2-[(2R)-4-(1H-benzimidazol-2-ylmethyl)-1-[2-fluoro-5-[(4-oxo-3H-phthalazin-1-yl)methyl]benzoyl]piperazin-2-yl]acetonitrile

Candidate 9 ranked third in the consensus docking evaluation, combining a supportive AutoDock Vina score (−7.509 kcal/mol) with a favorable Attracting Cavities SP-dG value (−7.9488 kcal/mol) (Table 2). Across its highest-confidence poses, the ligand consistently engaged LEU777/LEU778, ARG779, ASP783/ASP784, ASN793, GLU795, LYS796, and LYS798, indicating a pocket-consistent interaction pattern compatible with catalytic-site anchoring in PARP1.

Structurally, CID 172894737 represents a complementary phthalazinone-centered chemotype that combines a 4-oxo-3H-phthalazin-1-one pharmacophore with a benzimidazole-linked, nitrile-bearing piperazinyl side chain. This architecture is consistent with contemporary phthalazinone-derived PARP1 inhibitor design, where the phthalazinone core functions as a privileged anchoring motif and peripheral heterocyclic variation is used to tune potency, polarity, and developability [29,30]. The benzimidazole fragment adds an additional aromatic and polar recognition element, while the overall scaffold remains chemically simpler and potentially more tractable than the more heterocycle-dense, kinase-oriented architecture of Candidate 5 [31].

Within the present series, Candidate 9 occupies an intermediate position between the polarity-rich, solubility-oriented profile of Candidate 6 and the more mechanistically ambitious, dual-target-oriented profile of Candidate 5. Although it lacks explicit kinase-mimetic features associated with PARP1–PI3K design logic, its balanced polar–aromatic composition, moderate basicity, and phthalazinone anchoring pattern place it firmly within validated PARP1 inhibitor chemical space. Accordingly, CID 172894737 is best viewed as a scaffold-diversifying reserve chemotype that is mechanistically consistent with PARP1 binding and attractive for early-stage optimization. It should therefore be advanced to biochemical PARP1 confirmation, together with solubility and permeability profiling and assessment of PARP-trapping potential, which remains an important determinant of therapeutic relevance within phthalazinone-based PARP inhibitor series.

## 3. Discussion

This study establishes an interpretable ligand-based regression framework for PARP1 potency prediction using a curated ChEMBL dataset (n = 3339) and a deliberately reduced descriptor space (132 features from 1143 initial variables). The relatively weak GLM baseline (test R^2^ = 0.541) supports the view that PARP1 structure–activity relationships are nonlinear and interaction-rich, favoring flexible learners capable of capturing coupled fragment–property effects. Consistent with this, the stacked ensemble achieved the best held-out performance (test R^2^ = 0.723; RMSE = 0.610), indicating that blending RF, GB, ANN, and GLM improved generalization without materially sacrificing predictive signal. The train–test gap and mild compression at potency extremes are consistent with realistic out-of-sample behavior in heterogeneous bioactivity datasets rather than model instability.

A practical strength of the workflow is the Failure Mode Atlas, which translates aggregate performance into an operational reliability profile. On the held-out test set (n = 668), predictions were concentrated in the low-error regimes, with 83.7% of compounds within ≤0.75 pIC_50_ units and 97.3% within ≤1.5 pIC_50_ units. This supports a deployment strategy in which low-error predictions are prioritized for progression, whereas borderline or high-error cases are treated more cautiously and ideally supplemented by orthogonal evidence.

Interpretability was treated as a robustness criterion rather than a post hoc add-on. PFI and SHAP converged on a highly stable global driver set across training and test partitions, with both methods highlighting a compact core of Avalon fingerprint bits together with a constrained physicochemical envelope involving lipophilicity- and surface-related descriptors. This pattern is chemically plausible for PARP1, where productive binding depends on both privileged fragment motifs and an appropriate balance of polarity, surface partitioning, and scaffold topology.

External near-domain corroboration further supported the practical utility of the model. From the PubChem similarity expansion, 2067 hits with predicted PARP1 IC_50_ ≤ 10 nM and no measured PARP1 IC_50_ values were identified for prospective follow-up. Among similar PubChem inhibitors with retrievable PARP1 activity values, 366 compounds enabled direct comparison between predicted and reported activities. Although the variance explained on this external near-domain set was modest (R^2^ = 0.1240), the model retained useful rank-ordering and favorable absolute-error performance (RMSE = 0.4905 pIC_50_ units; MAE = 0.3295 pIC_50_ units), with 76.2% of compounds within ±0.5 pIC_50_ units and 95.4% within ±1.0 pIC_50_ units of experiment. These results indicate that the model is better suited to shortlist generation and triage than to exact recovery of the full experimental dynamic range across external assays.

To refine prospective candidates structurally, the final docking shortlist was constructed by ranking the 2067 PubChem hits by predicted potency and retaining the top Bemis–Murcko scaffold-diverse representatives. Ten structurally distinct ligands were then evaluated against PARP1 (4R6E) using two complementary docking layers performed through the SwissDock platform: exploratory AutoDock Vina and standardized Attracting Cavities (AC) 2.0. Because SwissDock imposes an approximate 10 min runtime cap for individual Vina jobs, exhaustiveness could not be fully identical across all exploratory Vina runs; therefore, Vina was treated as a supportive pose-ranking layer, whereas the fully standardized AC calculations formed the primary basis for final docking prioritization.

Under this consensus docking framework, CID 168873053, CID 175154210, and CID 172894737 emerged as the three most strongly supported ligands. In the standardized AC calculations, they yielded the most favorable SP-dG values (−8.5027, −8.0426, and −7.9488 kcal/mol, respectively), all more favorable than the niraparib reference ligand (−6.8549 kcal/mol). Their corresponding Vina scores (−7.486, −7.356, and −7.509 kcal/mol) were also supportive of stable pocket accommodation. CID 142736906 remained notable as a reserve candidate because it produced the most favorable exploratory Vina score (−8.107 kcal/mol) while still showing favorable AC support (−7.3332 kcal/mol). Across the selected ligands, the recovered poses repeatedly involved residues such as LEU778, ARG779, ASP783/ASP784, LYS796, LYS798, VAL679, GLY780, and ASN793, consistent with plausible accommodation in a PARP1-compatible binding environment.

Rather than claiming patent-aware filtering, the present analysis is more appropriately described as patent-contextualized. This is particularly relevant for CID 175154210, whose kinase-like heterocyclic side-chain architecture is more suggestive of a PARP1–PI3K dual-target rationale than the other top-ranked ligands. This interpretation remains qualitative and does not substitute for direct biochemical profiling or freedom-to-operate analysis.

Several limitations should be recognized. First, ChEMBL IC_50_ values aggregate across assays and conditions, so median aggregation reduces but does not eliminate label noise. Second, the PubChem corroboration set is near-domain, not truly out-of-domain, because it derives from high-similarity expansion around potent PARP1 seeds. Third, docking scores should be interpreted as relative pose-compatibility and ranking signals, not as experimental binding free energies. Finally, the current model targets biochemical potency only and does not directly encode clinically important properties such as PARP trapping, PARP1/PARP2 selectivity, or permeability/efflux liabilities. These limitations do not reduce the value of the workflow, but they define its appropriate use-case: mechanistically informed prioritization rather than direct therapeutic prediction.

## 4. Materials and Methods

This section delineates the computational framework developed to support robust, externally generalizable, and chemically interpretable prediction of PARP1 inhibitory potency. All analyses were executed within a fully reproducible, end-to-end workflow in the KNIME Analytics Platform (KNIME ver. 5.4), a modular, open-source environment that enables transparent, node-based construction of data-science pipelines while interoperating with external scripting engines and machine-learning backends [32]. RDKit served as the core cheminformatics layer, providing standardized molecular I/O, structure normalization, and the computation of physicochemical descriptors and fingerprints through a widely adopted open-source toolkit for chemical informatics [33]. Unless otherwise stated, all data-dependent transformations and feature-selection steps were derived exclusively from the training partition and then applied verbatim to the held-out test set and the external validation compounds, thereby enforcing strict separation between model development and evaluation, preventing information leakage, and yielding a realistic estimate of out-of-distribution generalization.

### 4.1. Data Acquisition and Curation

**Figure 6:**
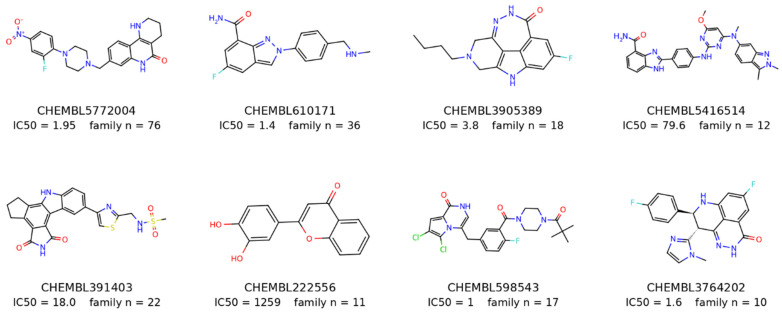
Representative ligands from the modeled PARP1 inhibitor dataset with reported IC50 values. Atom colors follow standard chemical drawing conventions: carbon, black/gray; nitrogen, blue; oxygen, red; fluorine, cyan; chlorine, green; sulfur, yellow.

A high-confidence PARP1 bioactivity dataset was assembled to support supervised regression modeling. In total, 3339 unique small molecules with experimentally reported PARP1 IC_50_ values were retrieved programmatically from ChEMBL v33, a curated, open-access repository of bioactive, drug-like compounds [34]. Figure 6 displays eight exemplar compounds with the lowest IC_50_ values, selected from the most frequent and structurally distinct Bemis–Murcko scaffold families in the curated PARP1 inhibitor dataset. Collectively, these representatives illustrate the chemical diversity captured by the ligand-based modeling set, whereas Figure S1 provides a broader scaffold-level overview of the most frequent Bemis–Murcko families among PARP1 inhibitors with reported IC_50_ ≤ 1000 nM. Each panel shows one representative compound together with its identifier, experimentally determined IC_50_ value, and the total number of molecules assigned to the corresponding scaffold family.

### 4.2. Quality Control and Filtering

To reduce assay-derived noise and maximize label reliability, the raw data were subjected to a stringent, multi-stage curation protocol:

Exact-value enforcement: Only records annotated with the relational operator “=” were retained; entries reported as inequalities (e.g., “>”, “<”, “≥”, “≤”) were excluded to avoid censoring effects.

Unit harmonization: All activity values were standardized to molarity (M) to ensure consistent downstream comparisons and transformations.

Assay confidence restriction: Only assays with ChEMBL confidence scores of 8–9 were included, reflecting high-confidence, direct target assignment supported by experimental evidence.

Duplicate resolution: Where multiple IC_50_ measurements existed for the same compound, duplicates were consolidated by taking the median IC_50_ to attenuate the influence of outliers and inter-assay variability. If only a single IC_50_ record was available, the most recent entry was retained to reflect updated experimental conditions and curation standards.

### 4.3. Endpoint Transformation

IC_50_ values were converted to pIC_50_ according to pIC_50_ = 9 − log_10_(IC_50_ [nM]). This transformation reduces scale skewness and places the activity values on a more modeling-suitable logarithmic scale. This corresponds to the conventional molar pIC_50_ up to a constant +9 shift and therefore preserves rank-ordering and relative differences.

### 4.4. Train–Test Partitioning and Leakage Control

Following curation, the dataset was stratified and randomly partitioned into an 80% training set (n = 2671) and a 20% independent test set (n = 668). The test set was held out as a strict final evaluation set and remained completely isolated throughout model development. Specifically, no preprocessing, descriptor scaling, feature selection, or hyperparameter optimization steps were informed by the test set at any stage, ensuring an unbiased estimate of generalization to unseen chemical space.

### 4.5. Molecular Representation and Descriptor Engineering

To capture both chemically interpretable physicochemical determinants and fragment-level structural signals relevant to PARP1 inhibition, standardized molecular structures were encoded using complementary descriptor families.

Structure standardization (RDKit v2023.09.1): All molecules were standardized using RDKit to ensure consistent representation and to minimize noise arising from inconsistent input structures. Standardization comprised aromaticity normalization (kekulization), valence and charge sanitization, salt/solvent removal by retaining the largest disconnected fragment, and tautomer canonicalization to reduce redundancy attributable to tautomeric multiplicity and to improve comparability across compounds.

Feature computation (total = 1143): For each standardized molecule, a combined feature vector was generated consisting of:

RDKit 2D descriptors (n = 119): Interpretable physicochemical and topological properties—including molecular weight (MW), octanol/water partition coefficient (logP), topological polar surface area (TPSA), hydrogen-bond donors/acceptors (HBD/HBA), and rotatable bond counts—selected for direct mechanistic interpretability and established relevance to structure–activity relationships (SAR).

Avalon fingerprints (1024-bit): A dictionary-based substructure encoding that represents the presence/absence of predefined fragments in a compact binary format. This representation offers broad structural coverage and is well-suited to capturing nonlinear SAR effects while remaining amenable to post hoc interpretation at the fragment level.

Rationale: The combined descriptor space was constructed to balance interpretability with structural resolution.

RDKit 2D physicochemical descriptors encode mechanistically meaningful properties—such as logP, TPSA, hydrogen-bonding capacity, and connectivity-derived topological indices—thereby supporting transparent SAR interpretation and providing a strong baseline for ligand-based QSAR when a consistent binding pose is unavailable or uncertain [35].

In parallel, Avalon fingerprints provide a dense, fragment-level, path-oriented hashed representation that captures subtle and distributed structural patterns across scaffolds. Their broad fragment coverage and moderate bit density facilitate nonlinear learners in modeling continuous potency trends with improved fidelity, particularly in chemically diverse datasets where activity may arise from multiple, partially overlapping substructural determinants. Avalon fingerprints have an established record of utility in QSAR, as originally described by Gedeck et al. [36].

Together, these complementary descriptors yield a chemically coherent feature space that supports both high-performance QSAR modeling and robust post hoc attribution analyses.

### 4.6. Preprocessing and Dimensionality Control

A controlled preprocessing pipeline was employed to mitigate redundancy, stabilize model training, and reduce the risk of overfitting in high-dimensional chemical space.

Leakage prevention: All thresholds and transformation parameters were derived solely from the training set and then applied unchanged to the test set and to external compounds.

Continuous-descriptor preprocessing: Z-score normalization (mean 0, standard deviation 1) for continuous descriptors;

Low-variance filtering removing descriptors with variance < 0.01;

Correlation filtering to reduce multicollinearity: To reduce multicollinearity in the descriptor space, a correlation-based filtering step was performed. Pearson correlation coefficients were computed for all features, and whenever two descriptors exceeded the predefined threshold (|r| > 0.90), one of the redundant variables was removed. This procedure retained only one representative descriptor from each highly correlated pair, thereby reducing redundancy and enhancing model robustness.

Outcome: After preprocessing, 1081 descriptors/fingerprint bits were retained as informative features, and the learned scaling and selection rules were propagated consistently to all subsequent datasets.

### 4.7. Supervised Feature Selection via Random Forest Importance

To obtain a parsimonious, activity-relevant feature set that enhances interpretability and generalization, supervised feature selection was performed using Random Forest (RF) variable importance measures [37].

Model and implementation: RF regression was implemented in H2O.ai ver. 3 through the KNIME H2O Machine Learning Integration, which supports distributed RF training and exposes model-derived variable-importance scores via the H2O Random Forest Learner node. This KNIME–H2O integration combines KNIME’s visual, workflow-based orchestration with H2O’s scalable learning engines, enabling efficient training and inspection of large nonlinear models within a single auditable pipeline [38]. The RF was configured with 500 trees and a maximum depth of 50 to capture nonlinear structure–activity relationships (SAR) and higher-order interactions typical of medicinal chemistry datasets.

Selection criterion: Features were ranked using the normalized importance scores produced by the fitted H2O RF model. Descriptors with importance values < 0.01 were removed. The KNIME H2O extension provides direct access to these importance values, enabling transparent and reproducible supervised filtering within the workflow.

Outcome and justification: This procedure yielded a final subset of 132 descriptors, providing a favorable bias–variance trade-off while retaining mechanistically meaningful structure–property signals relevant to PARP1 potency. RF-based ranking is particularly well suited to cheminformatics feature spaces containing mixed continuous descriptors and binary fingerprints, as it naturally models nonlinearities and feature interactions without requiring explicit functional-form assumptions or manual interaction engineering. This usage aligns with KNIME’s H2O workflow materials, which emphasize RF modeling and importance-driven evaluation in chemical datasets.

**Figure 7:**
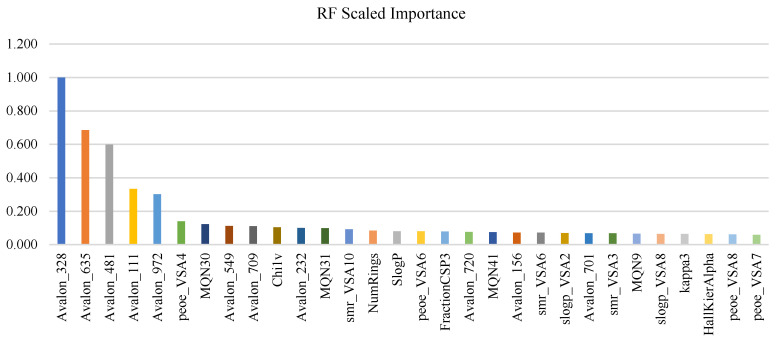
Top 30 RF scaled-importance features (normalized to Avalon_328 = 1.0), highlighting dominant fragment-level Avalon bits alongside key physicochemical/topological descriptors (e.g., PEOE_VSA4, MQN30, NumRings, slogP_VSA terms).

Figure 7 summarizes the top 30 RF scaled-importance features after supervised ranking, with the most influential variable (Avalon_328) normalized to 1.0. The ranking is led by multiple Avalon fingerprint bits (e.g., Avalon_635, Avalon_481, Avalon_111, Avalon_972), indicating that the RF model relies strongly on specific fragment-level motifs that recur among potent PARP1 chemotypes. This dominance is expected in target-focused QSAR because hashed fragment bits often encode binding-relevant substructures more directly than global physicochemical descriptors, which tend to be more general and less discriminative across closely related inhibitor series. Importantly, several interpretable RDKit-derived descriptors also appear among the leading features (e.g., PEOE_VSA4, MQN30, ChiV, NumRings, and multiple slogP_VSA terms), suggesting that potency is additionally shaped by global properties such as polar surface distribution, ring content, and lipophilicity. Applying the RF importance threshold (<0.01) reduced the representation to 132 features (~12% of the starting space), yielding a more parsimonious set that preserves both fragment-driven SAR signals and physicochemical constraints, thereby mitigating overfitting while retaining chemically meaningful determinants of PARP1 inhibitory activity.

### 4.8. Ensemble Development and Hyperparameter Optimization

A multi-model regression strategy was adopted to exploit complementary inductive biases across heterogeneous learners, culminating in a stacked ensemble that delivers robust predictions under chemical-space variability [39].

Base regressors. Four diverse regression algorithms were trained on the curated 132-feature representation:

Random Forest (RF): a bagged decision-tree ensemble well suited to nonlinear structure–activity relationships (SAR) and typically robust to noise and outliers. RF models were implemented using the H2O engine via the KNIME H2O Machine Learning Integration.

Generalized Linear Model (GLM): an interpretable linear baseline providing a calibrated, low-variance reference model.

Gradient Boosting Machine (GBM): an additive boosting framework that captures structured nonlinearities and hierarchical SAR patterns through sequential error correction.

Artificial Neural Network (ANN): a feed-forward multilayer architecture capable of learning subtle, distributed relationships in descriptor space that may not be well approximated by additive or piecewise-constant models.

Tuning protocol: Each base model was evaluated using 10-fold cross-validation with a randomized hyperparameter search. Random search was selected for its computational efficiency and strong empirical performance in high-dimensional hyperparameter spaces, where exhaustive grid exploration is often inefficient. During optimization, models were ranked using the Mean Residual Deviance (MRD), which is equivalent to the mean squared error (MSE) in regression. KNIME’s H2O integration provides native support for cross-validated model training and standardized evaluation, enabling scalable and reproducible model-selection workflows within the same provenance-tracked pipeline.

Stacked ensemble construction: The final predictive model was generated using H2O AutoML, which automatically constructs a stacked ensemble in which a GLM meta-learner integrates predictions from the best-performing base learners. H2O AutoML is explicitly designed to streamline high-quality ensemble construction through standardized training, automated scoring, and reproducible blending within KNIME’s visual workflow environment. By aggregating models with complementary error profiles, the stacked architecture reduces variance, improves generalization, and enhances resilience to chemical-space shift. The resulting ensemble is audit-ready due to H2O’s standardized AutoML procedures and KNIME’s transparent, end-to-end workflow provenance [39].

### 4.9. Reproducibility and Computational Environment

All analyses were executed within a single KNIME workflow to ensure procedural transparency and full reproducibility. The workflow integrated RDKit v2023.09.1 for structure standardization and descriptor computation and H2O.ai v3.42.0.3 for model training, cross-validation, AutoML stacking, and inference. To support deterministic regeneration of splits and model-tuning trajectories, the pipeline was executed with fixed random seeds for data partitioning and model training, where supported by the underlying libraries. All intermediate artifacts (curated datasets, feature matrices after each preprocessing stage, and trained model objects) were exported and versioned to enable exact re-running of the complete pipeline under the same software environment.

### 4.10. Data Leakage Control and Validation Hierarchy

Model development followed a strict validation hierarchy designed to prevent optimistic bias. Concretely: (i) all preprocessing and feature-selection decisions (normalization, variance/correlation filtering, and RF-based feature selection) were learned only on the training set; (ii) model hyperparameters were optimized using 10-fold cross-validation conducted within the training set; (iii) final performance was assessed once on the held-out independent test set, which remained untouched during model selection; and (iv) model transportability was further assessed by external validation on independently sourced PubChem compounds with reported experimental activity, transformed using the identical training-derived preprocessing and feature-selection rules. This sequence isolates model selection from evaluation and provides progressively stricter evidence of generalization across both chemical space and assay context.

### 4.11. Model Evaluation Metrics and Reporting Conventions

Model performance was quantified using a complementary suite of regression metrics consistent with cheminformatics reporting norms and best practice in predictive modeling. All primary metrics were computed using the H2O Numeric Scorer, which provides standardized evaluation of regression outputs in pIC_50_ units. The following metrics were used:

Mean Squared Error (MSE): the average squared deviation between predicted and experimental pIC_50_ values, providing a variance-weighted measure of predictive error.

Coefficient of Determination (R^2^): the proportion of variance in experimental pIC_50_ explained by the model, reflecting overall goodness-of-fit on a given evaluation split.

Root Mean Squared Error (RMSE): the square root of MSE, reported in pIC_50_ units to provide an interpretable error scale while retaining sensitivity to larger deviations.

Residual Deviance: for Gaussian regression in H2O, residual deviance is equivalent to MSE and was used as an additional standardized summary of residual dispersion and model fit.

Generalization under distribution shift: Beyond point-estimate accuracy, model assessment emphasized behavior under distribution shift (training → test → external). Performance gradients across these layers were interpreted as evidence of deployability rather than merely within-domain learnability, with particular attention to whether errors remained bounded when moving to chemically and experimentally shifted compounds.

Bias, error dispersion, and calibration-oriented diagnostics: Prediction quality was examined across the pIC_50_ range to identify systematic bias (consistent over- or under-prediction) and heteroscedastic error dispersion—both of which directly influence confidence in compound ranking during virtual screening. In this context, “calibration” was treated operationally as the absence of systematic residual structure and the maintenance of stable error statistics across potency regimes and validation layers.

Uncertainty handling (pragmatic): Uncertainty was addressed through ensemble robustness rather than explicit probabilistic modeling: the stacked ensemble aggregates learners with different inductive biases, and the consistency of predictions across the ensemble constituents served as an implicit reliability signal. In downstream prioritization, candidates exhibiting stable predicted potency and consistent performance behavior across validation layers were treated as higher-confidence selections for experimental triage.

### 4.12. PubChem Similarity Expansion and External Validation

To assess generalizability beyond the ChEMBL-derived calibration domain and to explore adjacent chemical space for prospective PARP1 inhibitors, we performed a high-stringency PubChem similarity expansion followed by QSAR ranking and orthogonal, consensus docking triage.

Similarity search and chemical-space expansion. Experimentally potent PARP1 inhibitors from the curated ChEMBL set (IC_50_ < 10 nM) were used as seed structures for PubChem similarity retrieval. Structural proximity was quantified by the Tanimoto coefficient, applying a stringent cutoff (Tanimoto > 0.90) to capture close analogs while still sampling local scaffold neighborhoods that may be underrepresented in the training distribution. This search yielded 32,450 structurally related compounds, defining a large but chemically coherent analog space for prospective screening.

Large-scale potency prediction and prioritization. All retrieved PubChem analogs were standardized using the same structure-processing and feature-transformation pipeline established for model training, then scored by the optimized stacked-ensemble QSAR model to obtain predicted pIC_50_ values. This enabled systematic ranking by predicted potency and facilitated the identification of recurring, potency-associated structural patterns within the expanded neighborhoods.

Docking-based plausibility triage and lead nomination. To prioritize candidates with no previously reported PARP1 activity in PubChem, the ten lowest-predicted IC_50_ compounds lacking PubChem PARP1 annotations were advanced to an orthogonal structure-based plausibility check using both AutoDock Vina and SwissDock (Attracting Cavities). Docking was used to assess pose compatibility with the PARP1 nicotinamide-binding cleft and the recovery of conserved interaction motifs, rather than to infer absolute binding free energies. Based on the convergence of QSAR prioritization and consensus-docking support, the three highest-ranking candidates were selected for detailed SAR interrogation and benchmarked against the crystallographic reference ligand 3JD (niraparib).

### 4.13. Molecular Docking and Structural Validation

To complement ligand-based QSAR prioritization with orthogonal structure-based evidence, molecular docking was performed using the SwissDock platform in two modes: AutoDock Vina and Attracting Cavities (AC) 2.0. Docking was used as a structural plausibility filter to assess whether QSAR-prioritized ligands could adopt PARP1-consistent poses within the catalytic pocket, rather than as a direct surrogate for experimental binding affinity [11].

The human PARP1 crystal structure 4R6E was used as the receptor. Docking was carried out under a consistent receptor and site definition using the prepared PARP1 structure (4r6e_modified.pdb) and a fixed search space centered on the co-crystallized ligand-binding region. For the SwissDock calculations, the search box was defined as 20 × 20 × 20 Å, centered at (−91, 23, 43). The co-crystallized ligand 3JD (niraparib) was included as an internal reference and re-docked under the same conditions for comparative benchmarking.

Exploratory AutoDock Vina runs were performed using SwissDock to obtain relative pose rankings and affinity-surrogate scores (kcal/mol). Because the SwissDock web server imposes a practical runtime limit of approximately 10 min per Vina job, exhaustiveness could not be kept fully identical across all ligands. In each case, search depth was increased as much as permitted within the server limit. Accordingly, Vina results were interpreted primarily as a supportive pose-ranking layer.

In parallel, Attracting Cavities 2.0 docking was performed using SwissDock under standardized settings, including medium sampling exhaustivity, 8 random initial conditions (RICs), and buried-cavity prioritization. Because these AC calculations were performed under the same protocol for all ligands, the resulting SwissDock/AC scores were used as the primary basis for cross-ligand comparison and final docking prioritization [40].

Docking outputs were analyzed qualitatively by examining pocket localization, geometric fit, and recovery of PARP1-consistent interaction patterns relative to the niraparib reference. Thus, agreement between AutoDock Vina and Attracting Cavities was interpreted conservatively as complementary support for structural compatibility and candidate prioritization.

### 4.14. Explainable Artificial Intelligence (XAI)

To ensure that the predictive pipeline supports chemically interpretable and actionable discovery, rather than only accurate ranking, two complementary explainability approaches were applied: Permutation Feature Importance (PFI) and SHapley Additive exPlanations (SHAP). Together, these methods provide a coherent interpretability layer spanning both global structure–activity relationship (SAR) drivers and molecule-specific attribution, thereby supporting mechanistic hypothesis generation for PARP1 inhibitory potency [41].

Permutation Feature Importance (PFI) was used to estimate global feature relevance in a model-agnostic manner by quantifying the loss in predictive performance after random permutation of an individual descriptor. This operation disrupts the statistical association between the feature and the endpoint while leaving the remaining feature space unchanged. Features that cause the greatest deterioration in performance are therefore interpreted as those most strongly relied upon by the model. In cheminformatics, PFI is particularly useful because SAR signals often arise from nonlinear interactions between physicochemical descriptors and fragment-level patterns, making PFI a practical high-level measure of model dependence without imposing assumptions about model architecture [42].

SHAP was then applied to complement this global analysis with additive, prediction-level attributions grounded in cooperative game theory [43]. At the local level, SHAP explains why a given compound is predicted to be more or less potent by identifying the descriptors that most strongly shift the prediction in either direction. At the global level, aggregated SHAP summaries highlight descriptors that exert consistent influence across the dataset, thereby revealing recurrent potency-driving patterns. SHAP has become a widely used interpretability framework in chemical machine learning and QSAR because it can uncover chemically meaningful structure–property relationships and improve trust in predictive models [44]. However, SHAP may be sensitive to descriptor correlation and model choice, particularly in feature spaces with residual multicollinearity. For this reason, SHAP was interpreted alongside the more model-agnostic PFI analysis to obtain a more robust and chemically credible explanation of the model’s behavior.

## 5. Conclusions

This work presents a reproducible and interpretable QSAR-to-docking workflow for PARP1 inhibitor prioritization, integrating descriptor reduction, ensemble regression, explainable AI, external near-domain corroboration, and complementary docking. The stacked ensemble achieved strong held-out performance (test R^2^ = 0.723; RMSE = 0.610) together with a practically favorable reliability profile. PFI and SHAP converged on a stable and chemically coherent set of activity drivers, supporting the mechanistic interpretability of the model.

External corroboration with 366 PubChem compounds showed that the model retained useful ranking and triage capability beyond the original training/test split, while complementary docking refined the final shortlist structurally. Under the consensus docking framework, CID 168873053, CID 175154210, and CID 172894737 emerged as the most promising candidates, with CID 142736906 retained as a high-interest reserve. Overall, the workflow provides a transparent and practically useful basis for experimental triage of PARP1 inhibitor candidates.

### Future Perspectives

Several extensions would strengthen the translational value of the present workflow. First, incorporation of applicability-domain analysis and formal uncertainty quantification would improve decision support, particularly for compounds in the higher-error regimes. Second, extending the framework beyond biochemical potency to include PARP-trapping propensity, isoform selectivity, and permeability/efflux-related properties would improve alignment with therapeutically relevant behavior. Third, the immediate experimental priority should be biochemical PARP1 IC_50_ confirmation for CID 168873053, CID 175154210, and CID 172894737, together with CID 142736906 as a reserve comparator, followed by cellular validation, PARP-trapping assessment, and solubility/permeability profiling. In particular, CID 175154210 warrants orthogonal PI3K profiling to test the patent-contextualized dual-target hypothesis suggested by its scaffold architecture.

## Figures and Tables

**Table 1:** Training and test performance of optimized regression models for PARP1 pIC_50_ prediction.

Training Results						Test Results				
Metric\Model	RF	GLM	GB	ANN	Stacked	RF	GLM	GB	ANN	Stacked
Mean Squared Error	0.074	0.532	0.166	0.149	0.077	0.390	0.617	0.407	0.420	0.372
R^2^	0.948	0.629	0.884	0.896	0.946	0.710	0.541	0.697	0.687	0.723
RMSE	0.272	0.729	0.407	0.386	0.277	0.625	0.785	0.638	0.648	0.610
Residual Deviances	0.074	0.532	0.166	0.149	0.077	0.390	0.617	0.407	0.420	0.372

Note: Under the Gaussian regression setting used in KNIME/H2O, Residual Deviance is numerically identical to MSE.

**Table 2:** Final consensus docking summary for Candidates 1–10 and the niraparib reference ligand.

Final Rank	Ligand	PubChem CID	Predicted IC_50_ (nM)	Vina Score (kcal/mol)	AC SP-dG (kcal/mol)	Main Residues in Top Poses *	Selected
1	Candidate 6	168873053	1.127623	−7.486	−8.5027	LEU778, ARG779, VAL679, ILE790, ASP784, GLU795, LYS796, LYS798	Yes
2	Candidate 5	175154210	1.117791	−7.356	−8.0426	LEU777/778, ARG779, ASP783/ASP784, TYR794, GLU795, LYS796, LYS798	Yes
3	Candidate 9	172894737	1.253607	−7.509	−7.9488	LEU777/778, ARG779, ASP783/ASP784, GLU795, LYS796, LYS798, ASN793	Yes
4	Candidate 3	142736906	1.081978	−8.107	−7.3332	ILE673, PHE677, VAL679, LEU778, ARG779, ASN793, ILE790, ASP784	Reserve/high-interest
5	Candidate 2	175360042	1.079779	−6.147	−7.2822	LYS674, PHE677, VAL679, LEU778, ASN793, LYS796, ASP783/ASP784	No
6	Candidate 8	150420374	1.237923	−5.343	−7.1011	LEU778, ARG779, ASP783/ASP784, TYR794, GLU795, THR799, GLU842	No
7	Candidate 7	126602454	1.166798	−6.986	−7.0573	PHE677, ASP678, LEU777, SER782, VAL792, LYS796, LYS798	No
8	Candidate 10	168206741	1.269705	−5.965	−6.8703	PHE677, LYS674, LEU778, ARG779, ASP783/ASP784, ASN793, LYS796	No
—	Reference (niraparib/3JD)	24958200	4.5 (pred.)	−7.266	−6.8549	VAL679, PHE677, ASP678, LYS674, LEU778, ARG779, GLY780, ASP783, VAL792, ASN793	Comparator
9	Candidate 1	71576509	0.903844	−5.837	−6.8529	PHE677, VAL679, LEU778, ARG779, ASP783/ASP784, ILE790, ASN793	No
10	Candidate 4	22266715	1.108243	−5.868	−6.7023	PHE677, LEU778, ASP783, ASN793, VAL679, LYS674	No

* Main residues in top poses refer to the key PARP1 pocket residues repeatedly identified around the highest-ranked ligand poses in the AutoDock Vina and Attracting Cavities docking analyses.

**Table 3:** Comparative SAR summary of the top three QSAR-prioritized hits versus niraparib (3JD).

Feature	CID 168873053 (Candidate 6)	CID 175154210 (Candidate 5)	CID 172894737 (Candidate 9)	Niraparib (3JD; CID 24958200)
Evidence level	Docking-supported	Docking-supported	Docking-supported	Crystallographic reference (4R6E)
Best Vina score (kcal/mol)	−7.486	−7.356	−7.509	−7.266 (re-docked under the same protocol)
Best AC SP-dG (kcal/mol)	−8.5027	−8.0426	−7.9488	−6.8549
Core scaffold	Phthalazinone	Phthalazinone	Phthalazinone	Indazole-carboxamide
Dominant structural profile	Most polarity-rich; heteroatom-dense	Kinase-like, nitrogen-rich appendages	Mixed polar–aromatic; benzimidazole-containing	Clinically optimized PARP inhibitor scaffold
Medicinal-chemistry interpretation	Most lead-like and potentially solubility-engineered	Most suggestive of dual-target PARP1–PI3K logic	Balanced reserve scaffold for diversification	Structural benchmark
Primary follow-up	PARP1 confirmation, trapping/residence-time assays, solubility and metabolic stability	PARP1 confirmation, PI3K profiling, early ADME/PK triage	PARP1 confirmation, solubility/permeability testing, trapping assessment	Reference for conserved pocket interactions
Overall role in the series	Top consensus hit	Mechanistically ambitious lead	Scaffold-diversifying reserve	Crystallographic comparator

Note: Vina scores and AC SP-dG values were obtained under the docking conditions summarized in Table 2. Final prioritization was weighted more heavily toward Attracting Cavities SP-dG, because the AC calculations were fully standardized across ligands, whereas the exploratory AutoDock Vina runs were subject to SwissDock runtime constraints.

## Data Availability

The original contributions presented in this study are included in the article/Supplementary Material. Further inquiries can be directed to the corresponding author.

## Supplementary Materials

The following supporting information can be downloaded at: https://www.mdpi.com/article/10.3390/ph19040584/s1. Table S1: PubChem compounds with both QSAR-predicted and experimentally reported PARP1 IC_50_ values. The experimental values correspond to the curated, best-measured PARP1 IC_50_ records extracted from PubChem assay summaries. Both IC_50_ and derived pIC_50_ values are provided for submission-ready reference. Figure S1: Most frequent Bemis–Murcko scaffold families among PARP1 inhibitors with reported IC_50_ ≤ 1000 nM. Figure S2: Stick-style views of the ligand–PARP1 complexes for the prioritized candidates and the reference ligand. Tables S2–S4: Docking shortlist and comparative AutoDock Vina and Attracting Cavities results.
