# Supplementary material for: Interpretable QSAR and Complementary Docking for PARP1 Inhibitor Prioritization: Reliability Stratification and Near-Domain Screening
Source: Pharmaceuticals (Basel). 2026 Apr 7;19(4):584. doi: 10.3390/ph19040584 (PMC13119234; doi:10.3390/ph19040584)
Supplement: Supplementary file 1 [file pharmaceuticals-19-00584-s001.zip › Table_S1_Predicted_PubChem_PARP1_IC50.pdf]

Supplementary Table S1

PubChem compounds with both QSAR-predicted and experimentally reported PARP1 IC50 values. Experimental values correspond to the curated best measured PARP1 IC50 record extracted from PubChem assay summaries. Both IC50 and derived pIC50 values are shown for submission-ready reference.

No.	CID	Pred. IC50 (nM)	Exp. IC50 (uM)	Exp. IC50 (nM)	Pred. pIC50	Exp. pIC50	AID(s)	PubMed ID(s)	PARP1 assay name(s)
1	164609478	0.4519	0.0002	0.1900	9.3450	9.7212	1764044	34091044	Inhibition of PARP1 (unknown origin)
2	155528446	0.4638	0.0004	0.4365	9.3337	9.3600	1581911	31846325	Inhibition of recombinant human PARP1 using histone as substrate after 1 hr in presence of biotinylated NAD+ by ELISA
3	155543524	0.4710	0.0007	0.6761	9.3270	9.1700	1581911	31846325	Inhibition of recombinant human PARP1 using histone as substrate after 1 hr in presence of biotinylated NAD+ by ELISA
4	164619328	0.4983	0.0002	0.2300	9.3025	9.6383	1764044	34091044	Inhibition of PARP1 (unknown origin)
5	168206990	0.5267	0.0004	0.4100	9.2785	9.3872	1876450	34656898	Inhibition of human PARP1 using NAD+ as substrate incubated for 1 hr by ELISA
6	164622937	0.5270	0.0005	0.4700	9.2782	9.3279	1764044	34091044	Inhibition of PARP1 (unknown origin)
7	155512651	0.5739	0.0006	0.6457	9.2411	9.1900	1581911	31846325	Inhibition of recombinant human PARP1 using histone as substrate after 1 hr in presence of biotinylated NAD+ by ELISA
8	141740038	0.6140	0.0005	0.4900	9.2118	9.3098	1764044	34091044	Inhibition of PARP1 (unknown origin)
9	164622316	0.6272	0.0005	0.5100	9.2026	9.2924	1764044	34091044	Inhibition of PARP1 (unknown origin)
10	155543234	0.6659	0.0007	0.6918	9.1766	9.1600	1581911	31846325	Inhibition of recombinant human PARP1 using histone as substrate after 1 hr in presence of biotinylated NAD+ by ELISA
11	145949586	0.6739	0.0001	0.1000	9.1714	10.0000	1499315	28692916	Inhibition of PARP1 (unknown origin) by ELISA
12	163196410	0.6963	0.0010	0.9600	9.1572	9.0177	1876450	34656898	Inhibition of human PARP1 using NAD+ as substrate incubated for 1 hr by ELISA
13	166021938	0.6994	0.0003	0.3400	9.1553	9.4685	1896630; 1896632	36343904	Inhibition of PARP-1 (unknown origin) | Inhibition of PARP-1 (unknown origin) binding to DNA assessed as DNA trapping activity
14	168293264	0.7708	0.0007	0.6600	9.1131	9.1805	1876450	34656898	Inhibition of human PARP1 using NAD+ as substrate incubated for 1 hr by ELISA
15	145959884	0.7781	0.0002	0.2000	9.1090	9.6990	1499315	28692916	Inhibition of PARP1 (unknown origin) by ELISA
16	145949467	0.7906	0.0558	55.8000	9.1020	7.2534	1499315	28692916	Inhibition of PARP1 (unknown origin) by ELISA
17	155511904	0.8208	0.0011	1.0960	9.0857	8.9602	1581911	31846325	Inhibition of recombinant human PARP1 using histone as substrate after 1 hr in presence of biotinylated NAD+ by ELISA
18	164623411	0.8345	0.0004	0.4000	9.0786	9.3979	1764044	34091044	Inhibition of PARP1 (unknown origin)
19	155562814	0.8630	0.0008	0.8318	9.0640	9.0800	1581911	31846325	Inhibition of recombinant human PARP1 using histone as substrate after 1 hr in presence of biotinylated NAD+ by ELISA
20	155513562	0.8706	0.0012	1.1750	9.0602	8.9300	1581911	31846325	Inhibition of recombinant human PARP1 using histone as substrate after 1 hr in presence of biotinylated NAD+ by ELISA
21	124081214	0.9101	0.0044	4.4000	9.0409	8.3565	1802336	27866910	In Vitro PARP1 Activity Assay from Article 10.1016/j.chembiol.2016.10.011: "Proteome-wide Profiling of Clinical PARP Inhibitors Reveals Compound-Specific Secondary Targets."
22	168294369	0.9162	0.0006	0.6100	9.0380	9.2147	1876450	34656898	Inhibition of human PARP1 using NAD+ as substrate incubated for 1 hr by ELISA
23	155512189	0.9401	0.0007	0.7244	9.0268	9.1400	1581911	31846325	Inhibition of recombinant human PARP1 using histone as substrate after 1 hr in presence of biotinylated NAD+ by ELISA
24	155528423	0.9469	0.0008	0.7800	9.0237	9.1079	1581911; 1872256	31846325; 35051747	Inhibition of recombinant human PARP1 using histone as substrate after 1 hr in presence of biotinylated NAD+ by ELISA | Inhibition of PARP1 (unknown origin) incubated for 1 hr by chemiluminescence assay
25	155555515	0.9739	0.0011	1.0720	9.0115	8.9698	1581911	31846325	Inhibition of recombinant human PARP1 using histone as substrate after 1 hr in presence of biotinylated NAD+ by ELISA

No.	CID	Pred. IC50 (nM)	Exp. IC50 (uM)	Exp. IC50 (nM)	Pred. pIC50	Exp. pIC50	AID(s)	PubMed ID(s)	PARP1 assay name(s)
26	126602436	0.9976	0.0009	0.9000	9.0011	9.0458	1682011	33120078	Inhibition of N-terminal GST-tagged human full length PARP1 (2 to 1041 residues) expressed in baculovirus infected Sf9 cells using histone mixture (H2A and H2B) and biotinylated NAD+ as substrate in presence of activated DNA incubated for 60 mins by chemiluminescence assay
27	145963753	1.0015	0.0009	0.9000	8.9994	9.0458	1499315	28692916	Inhibition of PARP1 (unknown origin) by ELISA
28	118679202	1.0382	0.0010	1.0000	8.9837	9.0000	1682011	33120078	Inhibition of N-terminal GST-tagged human full length PARP1 (2 to 1041 residues) expressed in baculovirus infected Sf9 cells using histone mixture (H2A and H2B) and biotinylated NAD+ as substrate in presence of activated DNA incubated for 60 mins by chemiluminescence assay
29	164620249	1.0693	0.0006	0.6300	8.9709	9.2007	1764044	34091044	Inhibition of PARP1 (unknown origin)
30	168284246	1.1104	0.0008	0.7800	8.9545	9.1079	1896630; 1896632	36343904	Inhibition of PARP-1 (unknown origin) | Inhibition of PARP-1 (unknown origin) binding to DNA assessed as DNA trapping activity
31	168282946	1.1208	0.0013	1.3100	8.9505	8.8827	1876450	34656898	Inhibition of human PARP1 using NAD+ as substrate incubated for 1 hr by ELISA
32	168268867	1.1341	0.0012	1.1900	8.9453	8.9245	1876450	34656898	Inhibition of human PARP1 using NAD+ as substrate incubated for 1 hr by ELISA
33	145962718	1.1570	0.0005	0.5000	8.9367	9.3010	1499315	28692916	Inhibition of PARP1 (unknown origin) by ELISA
34	126602424	1.1715	0.0011	1.1000	8.9313	8.9586	1682011	33120078	Inhibition of N-terminal GST-tagged human full length PARP1 (2 to 1041 residues) expressed in baculovirus infected Sf9 cells using histone mixture (H2A and H2B) and biotinylated NAD+ as substrate in presence of activated DNA incubated for 60 mins by chemiluminescence assay
35	172470594	1.1898	0.0135	13.4500	8.9245	7.8713	2012683	37605459	Inhibition of PARP1 (unknown origin) using histone as substrate by ELISA
36	118679197	1.1946	0.0006	0.6000	8.9228	9.2218	1682011	33120078	Inhibition of N-terminal GST-tagged human full length PARP1 (2 to 1041 residues) expressed in baculovirus infected Sf9 cells using histone mixture (H2A and H2B) and biotinylated NAD+ as substrate in presence of activated DNA incubated for 60 mins by chemiluminescence assay
37	145952366	1.2340	0.0009	0.9000	8.9087	9.0458	1499315	28692916	Inhibition of PARP1 (unknown origin) by ELISA
38	168272772	1.2538	0.0010	0.9800	8.9018	9.0088	1868336	35504210	Inhibition of PARP-1 (unknown origin) using biotinylated NAD+ as substrate incubated for 45 mins in the presence of deoxy-oligonucleotide by microplate reader method relative to control
39	126483592	1.2939	0.0006	0.6000	8.8881	9.2218	1682011	33120078	Inhibition of N-terminal GST-tagged human full length PARP1 (2 to 1041 residues) expressed in baculovirus infected Sf9 cells using histone mixture (H2A and H2B) and biotinylated NAD+ as substrate in presence of activated DNA incubated for 60 mins by chemiluminescence assay
40	126483567	1.2971	0.0004	0.4000	8.8870	9.3979	1682011	33120078	Inhibition of N-terminal GST-tagged human full length PARP1 (2 to 1041 residues) expressed in baculovirus infected Sf9 cells using histone mixture (H2A and H2B) and biotinylated NAD+ as substrate in presence of activated DNA incubated for 60 mins by chemiluminescence assay
41	145955549	1.3100	0.0004	0.4000	8.8827	9.3979	1499315	28692916	Inhibition of PARP1 (unknown origin) by ELISA
42	168278040	1.3360	0.0005	0.4900	8.8742	9.3098	1868336	35504210	Inhibition of PARP-1 (unknown origin) using biotinylated NAD+ as substrate incubated for 45 mins in the presence of deoxy-oligonucleotide by microplate reader method relative to control
43	168274891	1.3540	0.0015	1.5400	8.8684	8.8125	1876450	34656898	Inhibition of human PARP1 using NAD+ as substrate incubated for 1 hr by ELISA
44	126602483	1.3849	0.0010	1.0000	8.8586	9.0000	1682011	33120078	Inhibition of N-terminal GST-tagged human full length PARP1 (2 to 1041 residues) expressed in baculovirus infected Sf9 cells using histone mixture (H2A and H2B) and biotinylated NAD+ as substrate in presence of activated DNA incubated for 60 mins by chemiluminescence assay
45	168273464	1.4756	0.0016	1.6200	8.8310	8.7905	1868336	35504210	Inhibition of PARP-1 (unknown origin) using biotinylated NAD+ as substrate incubated for 45 mins in the presence of deoxy-oligonucleotide by microplate reader method relative to control
46	168271645	1.4921	0.0004	0.4100	8.8262	9.3872	1896630; 1896632	36343904	Inhibition of PARP-1 (unknown origin) | Inhibition of PARP-1 (unknown origin) binding to DNA assessed as DNA trapping activity
47	164609677	1.5002	0.0024	2.4000	8.8239	8.6198	1764044	34091044	Inhibition of PARP1 (unknown origin)
48	172457453	1.5041	0.0010	1.0200	8.8227	8.9914	2019975	37843892	Inhibition of human recombinant PARP1 expressed in Escherichia coli BL21(DE3) incubated for 1 hr by ELISA assay
49	145954997	1.5253	0.0006	0.6000	8.8166	9.2218	1499315	28692916	Inhibition of PARP1 (unknown origin) by ELISA
50	168283214	1.5863	0.0015	1.5400	8.7996	8.8125	1868336	35504210	Inhibition of PARP-1 (unknown origin) using biotinylated NAD+ as substrate incubated for 45 mins in the presence of deoxy-oligonucleotide by microplate reader method relative to control

No.	CID	Pred. IC50 (nM)	Exp. IC50 (uM)	Exp. IC50 (nM)	Pred. pIC50	Exp. pIC50	AID(s)	PubMed ID(s)	PARP1 assay name(s)
51	145949176	1.5946	0.0004	0.4000	8.7973	9.3979	1499315	28692916	Inhibition of PARP1 (unknown origin) by ELISA
52	25132952	1.6607	0.0020	2.0000	8.7797	8.6990	386691; 1798813	18800822	Inhibition of PARP1 by flashplate scintillation proximity assay | PARP-1 Enzyme Assay from Article 10.1021/jm8001263: "4-[3-(4-cyclopropanecarbonylpiperazine-1-carbonyl)-4-fluorobenzyl]-2H-phthalazin-1-one: a novel bioavailable inhibitor of poly(ADP-ribose) polymerase-1."
53	164616347	1.7247	0.0017	1.7000	8.7633	8.7696	1764044	34091044	Inhibition of PARP1 (unknown origin)
54	154402487	1.8063	0.0009	0.9000	8.7432	9.0458	1682011	33120078	Inhibition of N-terminal GST-tagged human full length PARP1 (2 to 1041 residues) expressed in baculovirus infected Sf9 cells using histone mixture (H2A and H2B) and biotinylated NAD+ as substrate in presence of activated DNA incubated for 60 mins by chemiluminescence assay
55	145951891	1.8260	0.0013	1.3000	8.7385	8.8861	1499315	28692916	Inhibition of PARP1 (unknown origin) by ELISA
56	92045137	1.8513	0.0010	1.0000	8.7325	9.0000	1265289	26546219	Inhibition of full length human PARP1 expressed in Baculovirus infected Sf9 insect cells using activated DNA as substrate after 1 hr by streptavidin-horseradish peroxidase-based luminescence assay
57	145962719	1.8769	0.0008	0.8000	8.7266	9.0969	1499315	28692916	Inhibition of PARP1 (unknown origin) by ELISA
58	162672785	1.8984	0.0026	2.6400	8.7216	8.5784	1697311	32924477	Inhibition of recombinant human C-terminal His-tagged PARP1 expressed in Sf9 insect cells preincubated for 20 mins in presence of activated DNA followed by 32P-NAD+ addition and further incubated for 2 hrs by chemiluminescence assay
59	168279253	1.9439	0.0029	2.9500	8.7113	8.5302	1862285	35780655	Binding affinity to PARP1 (unknown origin)
60	136186310	1.9633	0.0026	2.6300	8.7070	8.5800	1276413	26652717	Inhibition of human PARP1 using [3H]NAD as substrate after 1 min by microplate scintillation counting analysis
61	136100291	2.0034	0.0056	5.6000	8.6982	8.2518	1276413	26652717	Inhibition of human PARP1 using [3H]NAD as substrate after 1 min by microplate scintillation counting analysis
62	136385313	2.1130	0.0020	2.0200	8.6751	8.6946	1276413	26652717	Inhibition of human PARP1 using [3H]NAD as substrate after 1 min by microplate scintillation counting analysis
63	168206876	2.1652	0.0012	1.1700	8.6645	8.9318	1876450	34656898	Inhibition of human PARP1 using NAD+ as substrate incubated for 1 hr by ELISA
64	168282343	2.1775	0.0006	0.5900	8.6620	9.2291	1872256	35051747	Inhibition of PARP1 (unknown origin) incubated for 1 hr by chemiluminescence assay
65	164615614	2.2403	0.0020	2.0000	8.6497	8.6990	1759751	33859786	Inhibition of recombinant full length 6His-tagged PARP1 (unknown origin) incubated for 4 hrs by fluorescence anisotropy assay
66	169450724	2.2492	0.0009	0.9400	8.6480	9.0269	2019975; 2019981	37843892	Inhibition of human recombinant PARP1 expressed in Escherichia coli BL21(DE3) incubated for 1 hr by ELISA assay | Inhibition of human recombinant N-terminal GST-tagged PARP1 (2 to 1014(end) residues) expressed in Sf9 cells
67	168294528	2.2763	0.0013	1.3000	8.6428	8.8861	1896630; 1896632	36343904	Inhibition of PARP-1 (unknown origin) | Inhibition of PARP-1 (unknown origin) binding to DNA assessed as DNA trapping activity
68	168177317	2.2892	0.0006	0.5900	8.6403	9.2291	1896630; 1896632; 2060976	36343904	Inhibition of PARP-1 (unknown origin) | Inhibition of PARP-1 (unknown origin) binding to DNA assessed as DNA trapping activity | PARP-1 Enzyme Activity Assay from US Patent US20250034168: "NITROGEN-CONTAINING HETEROCYCLIC DERIVATIVE PARP INHIBITOR AND USE THEREOF"
69	145955534	2.2985	0.0000	0.0200	8.6386	10.6990	1499315	28692916	Inhibition of PARP1 (unknown origin) by ELISA
70	145956805	2.3221	0.0051	5.1000	8.6341	8.2924	1499315	28692916	Inhibition of PARP1 (unknown origin) by ELISA
71	156017220	2.4003	0.0018	1.8100	8.6197	8.7423	1667252	32088129	Inhibition of PARP1 (unknown origin)
72	156012470	2.4087	0.0026	2.5800	8.6182	8.5884	1667252	32088129	Inhibition of PARP1 (unknown origin)
73	136056842	2.4797	0.0033	3.2900	8.6056	8.4828	1276413	26652717	Inhibition of human PARP1 using [3H]NAD as substrate after 1 min by microplate scintillation counting analysis
74	136056880	2.5190	0.0025	2.4800	8.5988	8.6055	1276413	26652717	Inhibition of human PARP1 using [3H]NAD as substrate after 1 min by microplate scintillation counting analysis
75	126483601	2.5523	0.0008	0.8000	8.5931	9.0969	1682011	33120078	Inhibition of N-terminal GST-tagged human full length PARP1 (2 to 1041 residues) expressed in baculovirus infected Sf9 cells using histone mixture (H2A and H2B) and biotinylated NAD+ as substrate in presence of activated DNA incubated for 60 mins by chemiluminescence assay

No.	CID	Pred. IC50 (nM)	Exp. IC50 (uM)	Exp. IC50 (nM)	Pred. pIC50	Exp. pIC50	AID(s)	PubMed ID(s)	PARP1 assay name(s)
76	145954718	2.6170	0.0026	2.6000	8.5822	8.5850	1499315	28692916	Inhibition of PARP1 (unknown origin) by ELISA
77	172470399	2.6280	0.0021	2.0800	8.5804	8.6819	2019975	37843892	Inhibition of human recombinant PARP1 expressed in Escherichia coli BL21(DE3) incubated for 1 hr by ELISA assay
78	172471351	2.6396	0.0014	1.4000	8.5785	8.8539	2019975	37843892	Inhibition of human recombinant PARP1 expressed in Escherichia coli BL21(DE3) incubated for 1 hr by ELISA assay
79	155586926	2.6453	0.0020	2.0000	8.5775	8.6990	1759751	33859786	Inhibition of recombinant full length 6His-tagged PARP1 (unknown origin) incubated for 4 hrs by fluorescence anisotropy assay
80	44549376	2.6632	0.0022	2.2000	8.5746	8.6576	477514	19873981	Inhibition of human PARP1 by SPA
81	44549377	2.6632	0.0014	1.4000	8.5746	8.8539	477514; 1205269	19873981; 25761096	Inhibition of human PARP1 by SPA | Inhibition of PARP1 (unknown origin)
82	168177312	2.6703	0.0004	0.3600	8.5734	9.4437	1896630; 1896632; 2060976	36343904	Inhibition of PARP-1 (unknown origin) | Inhibition of PARP-1 (unknown origin) binding to DNA assessed as DNA trapping activity | PARP-1 Enzyme Activity Assay from US Patent US20250034168: "NITROGEN-CONTAINING HETEROCYCLIC DERIVATIVE PARP INHIBITOR AND USE THEREOF"
83	135924321	2.6713	0.0224	22.4000	8.5733	7.6498	1276413	26652717	Inhibition of human PARP1 using [3H]NAD as substrate after 1 min by microplate scintillation counting analysis
84	155560210	2.6902	0.0013	1.2900	8.5702	8.8894	1638275; 1949559	30684797; 27416328	Inhibition of PARP1 (unknown origin) using histone as substrate after 1 hr by ELISA | Inhibition of human PARP-1 using histone as substrate incubated for 16 hrs by ELISA assay
85	136242934	2.7507	0.0082	8.2000	8.5606	8.0862	1683890	33264017	Inhibition of human PARP-1 catalytic domain (662 to 1011 residues) expressed in Escherichia coli BL21(DE3) cells pre-incubated for 30 mins before addition of activated DNA and NAD by fluorescence based assay
86	56832570	2.7818	0.0017	1.7000	8.5557	8.7696	1557959	31401008	Inhibition of human PARP1 expressed in Escherichia coli incubated for 15 to 40 mins by ELISA
87	145959173	2.7936	0.0014	1.4000	8.5538	8.8539	1499315	28692916	Inhibition of PARP1 (unknown origin) by ELISA
88	164610419	2.7936	0.0015	1.5000	8.5538	8.8239	1758275	33740547	Inhibition of PARP1 (unknown origin) using biotin-NAD+ as substrate incubated for 1 hr by ELISA
89	155518789	2.8012	0.0011	1.1220	8.5527	8.9500	1581911	31846325	Inhibition of recombinant human PARP1 using histone as substrate after 1 hr in presence of biotinylated NAD+ by ELISA
90	172469120	2.8015	0.0027	2.7100	8.5526	8.5670	2019975	37843892	Inhibition of human recombinant PARP1 expressed in Escherichia coli BL21(DE3) incubated for 1 hr by ELISA assay
91	168177316	2.8324	0.0005	0.5100	8.5478	9.2924	1896630; 1896632; 1919942; 2060976	36343904	Inhibition of PARP-1 (unknown origin) | Inhibition of PARP-1 (unknown origin) binding to DNA assessed as DNA trapping activity | Biochemical (FP) Assay from US Patent US11802128: "Azetidine and pyrrolidine PARP1 inhibitors and uses thereof" | PARP-1 Enzyme Activity Assay from US Patent US20250034168: "NITROGEN-CONTAINING HETEROCYCLIC DERIVATIVE PARP INHIBITOR AND USE THEREOF"
92	145972651	2.8332	0.0008	0.8000	8.5477	9.0969	1499315	28692916	Inhibition of PARP1 (unknown origin) by ELISA
93	145955681	2.8860	0.0023	2.3000	8.5397	8.6383	1499315	28692916	Inhibition of PARP1 (unknown origin) by ELISA
94	136385224	2.9036	0.0020	2.0200	8.5371	8.6946	1276413	26652717	Inhibition of human PARP1 using [3H]NAD as substrate after 1 min by microplate scintillation counting analysis
95	145952328	2.9287	0.0024	2.4000	8.5333	8.6198	1499315	28692916	Inhibition of PARP1 (unknown origin) by ELISA
96	164622306	2.9309	0.0020	2.0000	8.5330	8.6990	1759751	33859786	Inhibition of recombinant full length 6His-tagged PARP1 (unknown origin) incubated for 4 hrs by fluorescence anisotropy assay
97	135924349	2.9572	0.0026	2.6000	8.5291	8.5850	1276413	26652717	Inhibition of human PARP1 using [3H]NAD as substrate after 1 min by microplate scintillation counting analysis
98	172454731	2.9587	0.0029	2.8500	8.5289	8.5452	2019975	37843892	Inhibition of human recombinant PARP1 expressed in Escherichia coli BL21(DE3) incubated for 1 hr by ELISA assay
99	164620063	2.9633	0.0009	0.9100	8.5282	9.0410	1758275	33740547	Inhibition of PARP1 (unknown origin) using biotin-NAD+ as substrate incubated for 1 hr by ELISA
100	46230374	2.9747	0.0035	3.5000	8.5266	8.4559	457780	20022747	Inhibition of PARP1
101	137238784	3.0338	0.0039	3.9000	8.5180	8.4089	1276413	26652717	Inhibition of human PARP1 using [3H]NAD as substrate after 1 min by microplate scintillation counting analysis

No.	CID	Pred. IC50 (nM)	Exp. IC50 (uM)	Exp. IC50 (nM)	Pred. pIC50	Exp. pIC50	AID(s)	PubMed ID(s)	PARP1 assay name(s)
102	156317994	3.0743	0.0010	1.0000	8.5123	9.0000	2202528		PARP-1 (poly[ADP-ribose] polymerase 1) Inhibitory Ability from US Patent US12459899: "Isoquinolinone derivatives, method for preparing the same, and pharmaceutical composition for preventing or treating poly(ADP-ribose) polymerase-1-related diseases, comprising the same as active ingredient"
103	137238783	3.1674	0.0029	2.8800	8.4993	8.5406	1276413	26652717	Inhibition of human PARP1 using [3H]NAD as substrate after 1 min by microplate scintillation counting analysis
104	71606889	3.1714	0.0018	1.8000	8.4988	8.7447	728437	23473053	Inhibition of human recombinant PARP1 after 1 hr by ELISA
105	172449106	3.1770	0.0030	3.0300	8.4980	8.5186	2019975	37843892	Inhibition of human recombinant PARP1 expressed in Escherichia coli BL21(DE3) incubated for 1 hr by ELISA assay
106	137238782	3.2085	0.0021	2.0800	8.4937	8.6819	1276413	26652717	Inhibition of human PARP1 using [3H]NAD as substrate after 1 min by microplate scintillation counting analysis
107	136186318	3.2098	0.0036	3.6300	8.4935	8.4401	1276413	26652717	Inhibition of human PARP1 using [3H]NAD as substrate after 1 min by microplate scintillation counting analysis
108	172442045	3.2512	0.0025	2.4600	8.4880	8.6091	2019975	37843892	Inhibition of human recombinant PARP1 expressed in Escherichia coli BL21(DE3) incubated for 1 hr by ELISA assay
109	46230509	3.2715	0.0028	2.8000	8.4853	8.5528	457780	20022747	Inhibition of PARP1
110	56833017	3.2717	0.0020	2.0000	8.4852	8.6990	1557958	31401008	Inhibition of human PARP1 expressed in Escherichia coli using histone as substrate by ELISA
111	155586931	3.2777	0.0030	3.0000	8.4844	8.5229	1759751	33859786	Inhibition of recombinant full length 6His-tagged PARP1 (unknown origin) incubated for 4 hrs by fluorescence anisotropy assay
112	145949294	3.2876	0.0035	3.5000	8.4831	8.4559	1499315	28692916	Inhibition of PARP1 (unknown origin) by ELISA
113	136186302	3.2892	0.0021	2.1400	8.4829	8.6696	1276413	26652717	Inhibition of human PARP1 using [3H]NAD as substrate after 1 min by microplate scintillation counting analysis
114	153525075	3.3009	0.0019	1.9400	8.4814	8.7122	1667252	32088129	Inhibition of PARP1 (unknown origin)
115	117955898	3.3054	0.0014	1.4000	8.4808	8.8539	2200719		Affinity Biochemical interaction: (inhibition of enzyme activity) EUB0002763a PARP1
116	145957654	3.3176	0.0054	5.4000	8.4792	8.2676	1499315	28692916	Inhibition of PARP1 (unknown origin) by ELISA
117	172457794	3.3453	0.0024	2.3800	8.4756	8.6234	2019975	37843892	Inhibition of human recombinant PARP1 expressed in Escherichia coli BL21(DE3) incubated for 1 hr by ELISA assay
118	164625133	3.3538	0.0030	2.9700	8.4745	8.5272	1758275	33740547	Inhibition of PARP1 (unknown origin) using biotin-NAD+ as substrate incubated for 1 hr by ELISA
119	146659481	3.3720	0.0039	3.8600	8.4721	8.4134	2019975	37843892	Inhibition of human recombinant PARP1 expressed in Escherichia coli BL21(DE3) incubated for 1 hr by ELISA assay
120	155587004	3.4238	0.0030	3.0000	8.4655	8.5229	1759751; 1895766	33859786; 34570508	Inhibition of recombinant full length 6His-tagged PARP1 (unknown origin) incubated for 4 hrs by fluorescence anisotropy assay | Inhibition of human recombinant N-terminal 6His-6Lys-TEV tagged PARP1 full length expressed in pFastBac expression system incubated for 4 hrs by fluorescence anisotropy binding assay
121	172440941	3.4555	0.0019	1.9100	8.4615	8.7190	2012683	37605459	Inhibition of PARP1 (unknown origin) using histone as substrate by ELISA
122	126483589	3.4594	0.0009	0.9000	8.4610	9.0458	1682011	33120078	Inhibition of N-terminal GST-tagged human full length PARP1 (2 to 1041 residues) expressed in baculovirus infected Sf9 cells using histone mixture (H2A and H2B) and biotinylated NAD+ as substrate in presence of activated DNA incubated for 60 mins by chemiluminescence assay
123	137632887	3.4626	0.0014	1.4000	8.4606	8.8539	1486467	28622906	Inhibition of recombinant human PARP-1 expressed in Escherichia coli BL21 (DE3) using sheared DNA as substrate in presence of biotinylated NAD after 1 hr by ELISA
124	155551208	3.4860	0.0095	9.5500	8.4577	8.0200	1581911; 1872256	31846325; 35051747	Inhibition of recombinant human PARP1 using histone as substrate after 1 hr in presence of biotinylated NAD+ by ELISA | Inhibition of PARP1 (unknown origin) incubated for 1 hr by chemiluminescence assay
125	135998415	3.4952	0.0024	2.4000	8.4565	8.6198	1276413	26652717	Inhibition of human PARP1 using [3H]NAD as substrate after 1 min by microplate scintillation counting analysis
126	136186312	3.5125	0.0040	4.0200	8.4544	8.3958	1276413	26652717	Inhibition of human PARP1 using [3H]NAD as substrate after 1 min by microplate scintillation counting analysis

No.	CID	Pred. IC50 (nM)	Exp. IC50 (uM)	Exp. IC50 (nM)	Pred. pIC50	Exp. pIC50	AID(s)	PubMed ID(s)	PARP1 assay name(s)
127	135924334	3.5297	0.0015	1.4600	8.4523	8.8356	1276413	26652717	Inhibition of human PARP1 using [3H]NAD as substrate after 1 min by microplate scintillation counting analysis
128	155513410	3.5599	0.0026	2.5700	8.4486	8.5901	1581911; 1872256	31846325; 35051747	Inhibition of recombinant human PARP1 using histone as substrate after 1 hr in presence of biotinylated NAD+ by ELISA | Inhibition of PARP1 (unknown origin) incubated for 1 hr by chemiluminescence assay
129	136242918	3.5710	0.0023	2.3000	8.4472	8.6383	1683890	33264017	Inhibition of human PARP-1 catalytic domain (662 to 1011 residues) expressed in Escherichia coli BL21(DE3) cells pre-incubated for 30 mins before addition of activated DNA and NAD by fluorescence based assay
130	172447047	3.5785	0.0030	3.0200	8.4463	8.5200	2019975	37843892	Inhibition of human recombinant PARP1 expressed in Escherichia coli BL21(DE3) incubated for 1 hr by ELISA assay
131	172456159	3.5846	0.0034	3.4500	8.4456	8.4622	2019975	37843892	Inhibition of human recombinant PARP1 expressed in Escherichia coli BL21(DE3) incubated for 1 hr by ELISA assay
132	172455642	3.6341	0.0049	4.9200	8.4396	8.3080	2019975	37843892	Inhibition of human recombinant PARP1 expressed in Escherichia coli BL21(DE3) incubated for 1 hr by ELISA assay
133	10450266	3.6372	0.0056	5.6000	8.4392	8.2518	492548	20364863	Inhibition of PARP1
134	156317961	3.6460	0.0004	0.4100	8.4382	9.3872	2202528		PARP-1 (poly[ADP-ribose] polymerase 1) Inhibitory Ability from US Patent US12459899: "Isoquinolinone derivatives, method for preparing the same, and pharmaceutical composition for preventing or treating poly(ADP-ribose) polymerase-1-related diseases, comprising the same as active ingredient"
135	44408006	3.6615	0.0050	5.0000	8.4363	8.3010	260968	16290932	Inhibitory activity against PARP1
136	136130844	3.6658	0.0026	2.6300	8.4358	8.5800	1276413	26652717	Inhibition of human PARP1 using [3H]NAD as substrate after 1 min by microplate scintillation counting analysis
137	168856754	3.6775	0.0032	3.1620	8.4344	8.5000	2038659	38116419	Inhibition of PARP1 (unknown origin) incubated for 4 hrs by fluorescent polarization assay
138	168856720	3.7017	0.0025	2.5120	8.4316	8.6000	2038659	38116419	Inhibition of PARP1 (unknown origin) incubated for 4 hrs by fluorescent polarization assay
139	25132954	3.7159	0.0030	3.0000	8.4299	8.5229	386691; 1798813	18800822	Inhibition of PARP1 by flashplate scintillation proximity assay | PARP-1 Enzyme Assay from Article 10.1021/jm8001263: "4-[3-(4-cyclopropanecarbonylpiperazine-1-carbonyl)-4-fluorobenzyl]-2H-phthalazin-1-one: a novel bioavailable inhibitor of poly(ADP-ribose) polymerase-1."
140	25133978	3.7304	0.0030	3.0000	8.4282	8.5229	386691; 1798813	18800822	Inhibition of PARP1 by flashplate scintillation proximity assay | PARP-1 Enzyme Assay from Article 10.1021/jm8001263: "4-[3-(4-cyclopropanecarbonylpiperazine-1-carbonyl)-4-fluorobenzyl]-2H-phthalazin-1-one: a novel bioavailable inhibitor of poly(ADP-ribose) polymerase-1."
141	126483579	3.7327	0.0006	0.6000	8.4280	9.2218	1682011	33120078	Inhibition of N-terminal GST-tagged human full length PARP1 (2 to 1041 residues) expressed in baculovirus infected Sf9 cells using histone mixture (H2A and H2B) and biotinylated NAD+ as substrate in presence of activated DNA incubated for 60 mins by chemiluminescence assay
142	155586901	3.7623	0.0003	0.3100	8.4245	9.5086	1895766; 1896630; 1896632	34570508; 36343904	Inhibition of human recombinant N-terminal 6His-6Lys-TEV tagged PARP1 full length expressed in pFastBac expression system incubated for 4 hrs by fluorescence anisotropy binding assay | Inhibition of PARP-1 (unknown origin) | Inhibition of PARP-1 (unknown origin) binding to DNA assessed as DNA trapping activity
143	135924322	3.7717	0.1360	136.0000	8.4235	6.8665	1276413	26652717	Inhibition of human PARP1 using [3H]NAD as substrate after 1 min by microplate scintillation counting analysis
144	136130845	3.7802	0.0024	2.3500	8.4225	8.6289	1276413	26652717	Inhibition of human PARP1 using [3H]NAD as substrate after 1 min by microplate scintillation counting analysis
145	156318318	3.8032	0.0008	0.7600	8.4199	9.1192	2202528		PARP-1 (poly[ADP-ribose] polymerase 1) Inhibitory Ability from US Patent US12459899: "Isoquinolinone derivatives, method for preparing the same, and pharmaceutical composition for preventing or treating poly(ADP-ribose) polymerase-1-related diseases, comprising the same as active ingredient"
146	164610509	3.8445	0.0088	8.8000	8.4152	8.0555	1758275	33740547	Inhibition of PARP1 (unknown origin) using biotin-NAD+ as substrate incubated for 1 hr by ELISA
147	46226377	3.8652	0.0040	4.0000	8.4128	8.3979	455679	20007017	Inhibition of human PARP1 after 3 hrs using [3H]NAD+ by scintillation proximity assay
148	172440832	3.8986	0.0027	2.7200	8.4091	8.5654	2019975	37843892	Inhibition of human recombinant PARP1 expressed in Escherichia coli BL21(DE3) incubated for 1 hr by ELISA assay

No.	CID	Pred. IC50 (nM)	Exp. IC50 (uM)	Exp. IC50 (nM)	Pred. pIC50	Exp. pIC50	AID(s)	PubMed ID(s)	PARP1 assay name(s)
149	172456013	3.9026	0.0037	3.6700	8.4086	8.4353	2019975	37843892	Inhibition of human recombinant PARP1 expressed in Escherichia coli BL21(DE3) incubated for 1 hr by ELISA assay
150	117734810	3.9410	0.0032	3.2000	8.4044	8.4949	1927623; 2200680; 2200893	32717529	Inhibition of PARP1 (unknown origin) | Affinity Biochemical interaction: (Inhibition of enzymatic activity, D+ used as substrate for PAR formation) EUB0002633aCl PARP1 | Affinity On-target Cellular interaction: (Inhibition of H2O2 induced PAR formation in Capan-1 cells) EUB0002633aCl PARP1
151	156318061	3.9574	0.0010	1.0000	8.4026	9.0000	2202528		PARP-1 (poly[ADP-ribose] polymerase 1) Inhibitory Ability from US Patent US12459899: "Isoquinolinone derivatives, method for preparing the same, and pharmaceutical composition for preventing or treating poly(ADP-ribose) polymerase-1-related diseases, comprising the same as active ingredient"
152	172439810	4.0055	0.0024	2.3900	8.3973	8.6216	2019975	37843892	Inhibition of human recombinant PARP1 expressed in Escherichia coli BL21(DE3) incubated for 1 hr by ELISA assay
153	168856718	4.0315	0.0040	3.9810	8.3945	8.4000	2038659	38116419	Inhibition of PARP1 (unknown origin) incubated for 4 hrs by fluorescent polarization assay
154	135875706	4.0372	0.0030	3.0000	8.3939	8.5229	367823; 1798825	19125579; 16288880	Inhibition of human recombinant PARP1 | PARP Enzyme Inhibition Assay from Article 10.1016/j.bmc.2005.09.061: "Discovery of potent and selective PARP-1 and PARP-2 inhibitors: SBDD analysis via a combination of X-ray structural study and homology modeling."
155	46226419	4.0379	0.0020	2.0000	8.3938	8.6990	455679	20007017	Inhibition of human PARP1 after 3 hrs using [3H]NAD+ by scintillation proximity assay
156	137238781	4.0512	0.0028	2.7600	8.3924	8.5591	1276413	26652717	Inhibition of human PARP1 using [3H]NAD as substrate after 1 min by microplate scintillation counting analysis
157	57394038	4.0776	0.0020	2.0000	8.3896	8.6990	638690	22153339	Inhibition of recombinant human PARP1 by in vitro assay
158	122190534	4.0935	0.0024	2.4160	8.3879	8.6169	1248040	26342868	Inhibition of PARP1 (unknown origin)
159	145977464	4.0973	0.0029	2.9000	8.3875	8.5376	1499315	28692916	Inhibition of PARP1 (unknown origin) by ELISA
160	155586982	4.1197	0.0020	2.0000	8.3851	8.6990	1806525		Fluorescence Anisotropy Binding Assay from US Patent US11325906: "Chemical compounds"
161	18444335	4.1342	0.0038	3.8000	8.3836	8.4202	241527; 367823	15837300; 19125579	Inhibitory concentration against human poly (ADP-ribose) polymerase 1 (PARP-1) | Inhibition of human recombinant PARP1
162	136242926	4.1615	0.0046	4.6000	8.3808	8.3372	1683890	33264017	Inhibition of human PARP-1 catalytic domain (662 to 1011 residues) expressed in Escherichia coli BL21(DE3) cells pre-incubated for 30 mins before addition of activated DNA and NAD by fluorescence based assay
163	136242925	4.1747	0.0045	4.5000	8.3794	8.3468	1683890	33264017	Inhibition of human PARP-1 catalytic domain (662 to 1011 residues) expressed in Escherichia coli BL21(DE3) cells pre-incubated for 30 mins before addition of activated DNA and NAD by fluorescence based assay
164	136344736	4.2050	0.0059	5.8500	8.3762	8.2328	1276413	26652717	Inhibition of human PARP1 using [3H]NAD as substrate after 1 min by microplate scintillation counting analysis
165	136385260	4.2050	0.0024	2.3600	8.3762	8.6271	1276413	26652717	Inhibition of human PARP1 using [3H]NAD as substrate after 1 min by microplate scintillation counting analysis
166	136385284	4.2050	0.0037	3.6500	8.3762	8.4377	1276413	26652717	Inhibition of human PARP1 using [3H]NAD as substrate after 1 min by microplate scintillation counting analysis
167	172456889	4.2079	0.0025	2.5400	8.3759	8.5952	2019975	37843892	Inhibition of human recombinant PARP1 expressed in Escherichia coli BL21(DE3) incubated for 1 hr by ELISA assay
168	46226396	4.2823	0.0014	1.4000	8.3683	8.8539	455679	20007017	Inhibition of human PARP1 after 3 hrs using [3H]NAD+ by scintillation proximity assay
169	166088917	4.3055	0.0007	0.6600	8.3660	9.1805	2060976; 2202408		PARP-1 Enzyme Activity Assay from US Patent US20250034168: "NITROGEN-CONTAINING HETEROCYCLIC DERIVATIVE PARP INHIBITOR AND USE THEREOF" | FP Assay from US Patent US20250289814: "COMPOUNDS AS PARP1 INHIBITIORS"
170	25133974	4.3087	0.0040	4.0000	8.3657	8.3979	386691; 1798813	18800822	Inhibition of PARP1 by flashplate scintillation proximity assay | PARP-1 Enzyme Assay from Article 10.1021/jm8001263: "4-[3-(4-cyclopropanecarbonylpiperazine-1-carbonyl)-4-fluorobenzyl]-2H-phthalazin-1-one: a novel bioavailable inhibitor of poly(ADP-ribose) polymerase-1."
171	25133634	4.3145	0.0050	5.0000	8.3651	8.3010	386691; 1798813	18800822	Inhibition of PARP1 by flashplate scintillation proximity assay | PARP-1 Enzyme Assay from Article 10.1021/jm8001263: "4-[3-(4-cyclopropanecarbonylpiperazine-1-carbonyl)-4-fluorobenzyl]-2H-phthalazin-1-one: a novel bioavailable inhibitor of poly(ADP-ribose) polymerase-1."

No.	CID	Pred. IC50 (nM)	Exp. IC50 (uM)	Exp. IC50 (nM)	Pred. pIC50	Exp. pIC50	AID(s)	PubMed ID(s)	PARP1 assay name(s)
172	136242900	4.3509	0.0033	3.3000	8.3614	8.4815	1683890	33264017	Inhibition of human PARP-1 catalytic domain (662 to 1011 residues) expressed in Escherichia coli BL21(DE3) cells pre-incubated for 30 mins before addition of activated DNA and NAD by fluorescence based assay
173	136242923	4.4305	0.0057	5.7000	8.3535	8.2441	1683890	33264017	Inhibition of human PARP-1 catalytic domain (662 to 1011 residues) expressed in Escherichia coli BL21(DE3) cells pre-incubated for 30 mins before addition of activated DNA and NAD by fluorescence based assay
174	25133977	4.4432	0.0020	2.0000	8.3523	8.6990	386691; 1798813	18800822	Inhibition of PARP1 by flashplate scintillation proximity assay | PARP-1 Enzyme Assay from Article 10.1021/jm8001263: "4-[3-(4-cyclopropanecarbonylpiperazine-1-carbonyl)-4-fluorobenzyl]-2H-phthalazin-1-one: a novel bioavailable inhibitor of poly(ADP-ribose) polymerase-1."
175	166021853	4.4442	0.0026	2.5800	8.3522	8.5884	2061048		PARP1/PARP2 Trapping Assay from US Patent US20250051324: "PARP INHIBITOR, PHARMACEUTICAL COMPOSITION COMPRISING SAME, AND USE THEREOF"
176	162524503	4.4735	0.1670	167.0000	8.3493	6.7773	2061152		PARP Fluorescence Anisotropy Binding Assay from US Patent US20250082630: "METHODS OF TREATING BRAIN TUMOURS AND NEUROBLASTOMAS"
177	164617022	4.4847	0.0019	1.9000	8.3483	8.7212	1758275	33740547	Inhibition of PARP1 (unknown origin) using biotin-NAD+ as substrate incubated for 1 hr by ELISA
178	172468369	4.5049	0.0090	9.0000	8.3463	8.0458	2019975	37843892	Inhibition of human recombinant PARP1 expressed in Escherichia coli BL21(DE3) incubated for 1 hr by ELISA assay
179	168277693	4.5549	0.0041	4.0800	8.3415	8.3893	1868336	35504210	Inhibition of PARP-1 (unknown origin) using biotinylated NAD+ as substrate incubated for 45 mins in the presence of deoxy-oligonucleotide by microplate reader method relative to control
180	145971228	4.5549	0.0097	9.7000	8.3415	8.0132	1499315	28692916	Inhibition of PARP1 (unknown origin) by ELISA
181	166021887	4.5642	0.0079	7.9000	8.3406	8.1024	1919321		Biochemical (FP) Assay from US Patent US11591331: "PARP1 inhibitors and uses thereof"
182	155531582	4.5966	0.0155	15.4900	8.3376	7.8099	1581911	31846325	Inhibition of recombinant human PARP1 using histone as substrate after 1 hr in presence of biotinylated NAD+ by ELISA
183	127034750	4.6068	0.0030	3.0000	8.3366	8.5229	1261692	26469301	Inhibition of PARP1 (unknown origin) incubated for 10 mins using biotinylated NAD+ and activated DNA by colorimetric assay
184	136242927	4.6308	0.0038	3.8000	8.3343	8.4202	1683890	33264017	Inhibition of human PARP-1 catalytic domain (662 to 1011 residues) expressed in Escherichia coli BL21(DE3) cells pre-incubated for 30 mins before addition of activated DNA and NAD by fluorescence based assay
185	168297855	4.6332	0.0020	2.0000	8.3341	8.6990	1866927	35306814	Inhibition of PARP1 (unknown origin)
186	136242917	4.6618	0.0033	3.3000	8.3314	8.4815	1683890	33264017	Inhibition of human PARP-1 catalytic domain (662 to 1011 residues) expressed in Escherichia coli BL21(DE3) cells pre-incubated for 30 mins before addition of activated DNA and NAD by fluorescence based assay
187	166021865	4.7214	0.0066	6.6000	8.3259	8.1805	1919321		Biochemical (FP) Assay from US Patent US11591331: "PARP1 inhibitors and uses thereof"
188	25133280	4.7445	0.0050	5.0000	8.3238	8.3010	386691; 1798813	18800822	Inhibition of PARP1 by flashplate scintillation proximity assay | PARP-1 Enzyme Assay from Article 10.1021/jm8001263: "4-[3-(4-cyclopropanecarbonylpiperazine-1-carbonyl)-4-fluorobenzyl]-2H-phthalazin-1-one: a novel bioavailable inhibitor of poly(ADP-ribose) polymerase-1."
189	71488522	4.7504	0.0032	3.2000	8.3233	8.4949	1638260	30684797	Inhibition of recombinant human PARP1 using histone as substrate after 1 hr in presence of NAD+ by ELISA
190	71626523	4.7728	0.0029	2.9000	8.3212	8.5376	1683890	33264017	Inhibition of human PARP-1 catalytic domain (662 to 1011 residues) expressed in Escherichia coli BL21(DE3) cells pre-incubated for 30 mins before addition of activated DNA and NAD by fluorescence based assay
191	162524552	4.7763	0.0070	7.0000	8.3209	8.1549	2061152		PARP Fluorescence Anisotropy Binding Assay from US Patent US20250082630: "METHODS OF TREATING BRAIN TUMOURS AND NEUROBLASTOMAS"
192	137648174	4.7852	0.0031	3.1000	8.3201	8.5086	1486467	28622906	Inhibition of recombinant human PARP-1 expressed in Escherichia coli BL21 (DE3) using sheared DNA as substrate in presence of biotinylated NAD after 1 hr by ELISA

No.	CID	Pred. IC50 (nM)	Exp. IC50 (uM)	Exp. IC50 (nM)	Pred. pIC50	Exp. pIC50	AID(s)	PubMed ID(s)	PARP1 assay name(s)
193	25133278	4.7883	0.0060	6.0000	8.3198	8.2218	386691; 1798813	18800822	Inhibition of PARP1 by flashplate scintillation proximity assay | PARP-1 Enzyme Assay from Article 10.1021/jm8001263: "4-[3-(4-cyclopropanecarbonylpiperazine-1-carbonyl)-4-fluorobenzyl]-2H-phthalazin-1-one: a novel bioavailable inhibitor of poly(ADP-ribose) polymerase-1."
194	44408065	4.8100	0.0050	5.0000	8.3179	8.3010	260968	16290932	Inhibitory activity against PARP1
195	46230327	4.8337	0.0057	5.7000	8.3157	8.2441	457780	20022747	Inhibition of PARP1
196	155555707	4.8372	0.0079	7.9430	8.3154	8.1000	1581911	31846325	Inhibition of recombinant human PARP1 using histone as substrate after 1 hr in presence of biotinylated NAD+ by ELISA
197	172468108	4.8407	0.0043	4.3300	8.3151	8.3635	2019975	37843892	Inhibition of human recombinant PARP1 expressed in Escherichia coli BL21(DE3) incubated for 1 hr by ELISA assay
198	46226418	4.8594	0.0060	6.0000	8.3134	8.2218	455679	20007017	Inhibition of human PARP1 after 3 hrs using [3H]NAD+ by scintillation proximity assay
199	72201273	4.8957	0.2000	200.0000	8.3102	6.6990	1872264	35051747	Inhibition of PARP1 (unknown origin) by ELISA
200	162524602	4.9232	0.0600	60.0000	8.3078	7.2218	2061152		PARP Fluorescence Anisotropy Binding Assay from US Patent US20250082630: "METHODS OF TREATING BRAIN TUMOURS AND NEUROBLASTOMAS"
201	172458938	4.9266	0.0038	3.7900	8.3075	8.4214	2019975	37843892	Inhibition of human recombinant PARP1 expressed in Escherichia coli BL21(DE3) incubated for 1 hr by ELISA assay
202	130292584	4.9293	0.0045	4.5200	8.3072	8.3449	1830902	34748333	Inhibition of recombinant human PARP1 using NAD+ as substrate incubated for 1 hr by ELISA
203	156318848	4.9346	0.0010	1.0000	8.3067	9.0000	2202528		PARP-1 (poly[ADP-ribose] polymerase 1) Inhibitory Ability from US Patent US12459899: "Isoquinolinone derivatives, method for preparing the same, and pharmaceutical composition for preventing or treating poly(ADP-ribose) polymerase-1-related diseases, comprising the same as active ingredient"
204	156317918	4.9372	0.0010	1.0000	8.3065	9.0000	2202528		PARP-1 (poly[ADP-ribose] polymerase 1) Inhibitory Ability from US Patent US12459899: "Isoquinolinone derivatives, method for preparing the same, and pharmaceutical composition for preventing or treating poly(ADP-ribose) polymerase-1-related diseases, comprising the same as active ingredient"
205	162666366	4.9519	0.0047	4.7000	8.3052	8.3279	1683890	33264017	Inhibition of human PARP-1 catalytic domain (662 to 1011 residues) expressed in Escherichia coli BL21(DE3) cells pre-incubated for 30 mins before addition of activated DNA and NAD by fluorescence based assay
206	162524431	4.9545	0.0150	15.0000	8.3050	7.8239	2061152		PARP Fluorescence Anisotropy Binding Assay from US Patent US20250082630: "METHODS OF TREATING BRAIN TUMOURS AND NEUROBLASTOMAS"
207	44549777	4.9573	0.0021	2.1000	8.3048	8.6778	477514	19873981	Inhibition of human PARP1 by SPA
208	136186303	4.9842	0.0053	5.2700	8.3024	8.2782	1276413	26652717	Inhibition of human PARP1 using [3H]NAD as substrate after 1 min by microplate scintillation counting analysis
209	156319006	5.0233	0.0006	0.6500	8.2990	9.1871	2202528		PARP-1 (poly[ADP-ribose] polymerase 1) Inhibitory Ability from US Patent US12459899: "Isoquinolinone derivatives, method for preparing the same, and pharmaceutical composition for preventing or treating poly(ADP-ribose) polymerase-1-related diseases, comprising the same as active ingredient"
210	25133633	5.0435	0.0060	6.0000	8.2973	8.2218	386691; 1798813	18800822	Inhibition of PARP1 by flashplate scintillation proximity assay | PARP-1 Enzyme Assay from Article 10.1021/jm8001263: "4-[3-(4-cyclopropanecarbonylpiperazine-1-carbonyl)-4-fluorobenzyl]-2H-phthalazin-1-one: a novel bioavailable inhibitor of poly(ADP-ribose) polymerase-1."
211	71626525	5.0552	0.0033	3.3000	8.2963	8.4815	1683890	33264017	Inhibition of human PARP-1 catalytic domain (662 to 1011 residues) expressed in Escherichia coli BL21(DE3) cells pre-incubated for 30 mins before addition of activated DNA and NAD by fluorescence based assay
212	46191765	5.0555	0.0040	4.0000	8.2962	8.3979	455679	20007017	Inhibition of human PARP1 after 3 hrs using [3H]NAD+ by scintillation proximity assay
213	146688721	5.0963	0.0047	4.6800	8.2927	8.3298	2019975	37843892	Inhibition of human recombinant PARP1 expressed in Escherichia coli BL21(DE3) incubated for 1 hr by ELISA assay
214	155546205	5.1045	0.0062	6.1660	8.2920	8.2100	1581911	31846325	Inhibition of recombinant human PARP1 using histone as substrate after 1 hr in presence of biotinylated NAD+ by ELISA
215	11725479	5.1577	0.0050	5.0000	8.2875	8.3010	241527; 367823; 1927623	15837300; 19125579; 32717529	Inhibitory concentration against human poly (ADP-ribose) polymerase 1 (PARP-1) | Inhibition of human recombinant PARP1 | Inhibition of PARP1 (unknown origin)

No.	CID	Pred. IC50 (nM)	Exp. IC50 (uM)	Exp. IC50 (nM)	Pred. pIC50	Exp. pIC50	AID(s)	PubMed ID(s)	PARP1 assay name(s)
216	71604565	5.1695	0.0450	45.0000	8.2865	7.3468	728437	23473053	Inhibition of human recombinant PARP1 after 1 hr by ELISA
217	11675005	5.2013	0.0041	4.1000	8.2839	8.3872	241527; 367823	15837300; 19125579	Inhibitory concentration against human poly (ADP-ribose) polymerase 1 (PARP-1) | Inhibition of human recombinant PARP1
218	130293051	5.2039	0.0008	0.8300	8.2837	9.0809	1830902	34748333	Inhibition of recombinant human PARP1 using NAD+ as substrate incubated for 1 hr by ELISA
219	44549378	5.2275	0.0031	3.1000	8.2817	8.5086	477514; 1205269	19873981; 25761096	Inhibition of human PARP1 by SPA | Inhibition of PARP1 (unknown origin)
220	156332857	5.2277	0.0008	0.8500	8.2817	9.0706	2202528		PARP-1 (poly[ADP-ribose] polymerase 1) Inhibitory Ability from US Patent US12459899: "Isoquinolinone derivatives, method for preparing the same, and pharmaceutical composition for preventing or treating poly(ADP-ribose) polymerase-1-related diseases, comprising the same as active ingredient"
221	156319379	5.2559	0.0006	0.6500	8.2794	9.1871	2202528		PARP-1 (poly[ADP-ribose] polymerase 1) Inhibitory Ability from US Patent US12459899: "Isoquinolinone derivatives, method for preparing the same, and pharmaceutical composition for preventing or treating poly(ADP-ribose) polymerase-1-related diseases, comprising the same as active ingredient"
222	156318298	5.2833	0.0006	0.6500	8.2771	9.1871	2202528		PARP-1 (poly[ADP-ribose] polymerase 1) Inhibitory Ability from US Patent US12459899: "Isoquinolinone derivatives, method for preparing the same, and pharmaceutical composition for preventing or treating poly(ADP-ribose) polymerase-1-related diseases, comprising the same as active ingredient"
223	137189936	5.2993	0.0076	7.6200	8.2758	8.1180	1276413	26652717	Inhibition of human PARP1 using [3H]NAD as substrate after 1 min by microplate scintillation counting analysis
224	145956391	5.3026	0.0112	11.2000	8.2755	7.9508	1499315	28692916	Inhibition of PARP1 (unknown origin) by ELISA
225	46226421	5.3533	0.0050	5.0000	8.2714	8.3010	455679	20007017	Inhibition of human PARP1 after 3 hrs using [3H]NAD+ by scintillation proximity assay
226	46230280	5.3648	0.0018	1.8000	8.2704	8.7447	457780	20022747	Inhibition of PARP1
227	168271990	5.3811	0.0028	2.7700	8.2691	8.5575	1849629	33129590	Inhibition of human full length PARP1 expressed in Escherichia coli rosetta (DE3) incubated for 20 mins by fluorescence analysis
228	25133632	5.4025	0.0060	6.0000	8.2674	8.2218	386691; 1798813	18800822	Inhibition of PARP1 by flashplate scintillation proximity assay | PARP-1 Enzyme Assay from Article 10.1021/jm8001263: "4-[3-(4-cyclopropanecarbonylpiperazine-1-carbonyl)-4-fluorobenzyl]-2H-phthalazin-1-one: a novel bioavailable inhibitor of poly(ADP-ribose) polymerase-1."
229	118905549	5.4334	0.0039	3.9000	8.2649	8.4089	1261692	26469301	Inhibition of PARP1 (unknown origin) incubated for 10 mins using biotinylated NAD+ and activated DNA by colorimetric assay
230	145957773	5.4398	0.0061	6.1000	8.2644	8.2147	1499315	28692916	Inhibition of PARP1 (unknown origin) by ELISA
231	73051683	5.4861	0.0043	4.3000	8.2607	8.3665	2138123	37567056	Inhibition of PARP1 (unknown origin) incubated for 1 hr by ELISA analysis
232	137636391	5.5036	0.0057	5.7000	8.2594	8.2441	1486467	28622906	Inhibition of recombinant human PARP-1 expressed in Escherichia coli BL21 (DE3) using sheared DNA as substrate in presence of biotinylated NAD after 1 hr by ELISA
233	156318991	5.5187	0.0010	1.0000	8.2582	9.0000	2202528		PARP-1 (poly[ADP-ribose] polymerase 1) Inhibitory Ability from US Patent US12459899: "Isoquinolinone derivatives, method for preparing the same, and pharmaceutical composition for preventing or treating poly(ADP-ribose) polymerase-1-related diseases, comprising the same as active ingredient"
234	169110636	5.5246	0.0030	3.0000	8.2577	8.5229	2006901	38352834	Displacement of Olaparib-BDY FL from PARP1 (unknown origin) incubated for 4 hrs by FP assay
235	136242916	5.5415	0.0051	5.1000	8.2564	8.2924	1683890	33264017	Inhibition of human PARP-1 catalytic domain (662 to 1011 residues) expressed in Escherichia coli BL21(DE3) cells pre-incubated for 30 mins before addition of activated DNA and NAD by fluorescence based assay
236	172448814	5.5592	0.0038	3.8100	8.2550	8.4191	2019975	37843892	Inhibition of human recombinant PARP1 expressed in Escherichia coli BL21(DE3) incubated for 1 hr by ELISA assay
237	46226397	5.5861	0.0200	20.0000	8.2529	7.6990	455679	20007017	Inhibition of human PARP1 after 3 hrs using [3H]NAD+ by scintillation proximity assay
238	172456644	5.6023	0.0097	9.7500	8.2516	8.0110	2019975	37843892	Inhibition of human recombinant PARP1 expressed in Escherichia coli BL21(DE3) incubated for 1 hr by ELISA assay
239	44407895	5.6047	0.0070	7.0000	8.2514	8.1549	260968	16290932	Inhibitory activity against PARP1
240	168177315	5.6166	0.0004	0.4100	8.2505	9.3872	2060976		PARP-1 Enzyme Activity Assay from US Patent US20250034168: "NITROGEN-CONTAINING HETEROCYCLIC DERIVATIVE PARP INHIBITOR AND USE THEREOF"

No.	CID	Pred. IC50 (nM)	Exp. IC50 (uM)	Exp. IC50 (nM)	Pred. pIC50	Exp. pIC50	AID(s)	PubMed ID(s)	PARP1 assay name(s)
241	10980119	5.6378	0.0050	5.0000	8.2489	8.3010	344541; 1798824	18713665	Inhibition of PARP1 in human HeLa cells by fluid scintillation counting using [adenylated-32P]NAD as substrate | PARP-1 Enzyme Inhibition Assay from Article 10.1016/j.bmcl.2008.07.091: "Identification of ring-fused pyrazolo pyridin-2-ones as novel poly(ADP-ribose)polymerase-1 inhibitors."
242	156317907	5.6383	0.0010	1.0000	8.2489	9.0000	2202528		PARP-1 (poly[ADP-ribose] polymerase 1) Inhibitory Ability from US Patent US12459899: "Isoquinolinone derivatives, method for preparing the same, and pharmaceutical composition for preventing or treating poly(ADP-ribose) polymerase-1-related diseases, comprising the same as active ingredient"
243	156317929	5.6383	0.0010	1.0000	8.2489	9.0000	2202528		PARP-1 (poly[ADP-ribose] polymerase 1) Inhibitory Ability from US Patent US12459899: "Isoquinolinone derivatives, method for preparing the same, and pharmaceutical composition for preventing or treating poly(ADP-ribose) polymerase-1-related diseases, comprising the same as active ingredient"
244	146479479	5.6481	0.1000	100.0000	8.2481	7.0000	1872263	35051747	Inhibition of N-terminal GST-tagged recombinant human PARP1 (2 to 1014 residues) expressed in Sf9 insect cells incubated for 1 hr by colorimetric assay
245	156318120	5.6502	0.0010	1.0000	8.2479	9.0000	2202528		PARP-1 (poly[ADP-ribose] polymerase 1) Inhibitory Ability from US Patent US12459899: "Isoquinolinone derivatives, method for preparing the same, and pharmaceutical composition for preventing or treating poly(ADP-ribose) polymerase-1-related diseases, comprising the same as active ingredient"
246	156318126	5.6502	0.0010	1.0000	8.2479	9.0000	2202528		PARP-1 (poly[ADP-ribose] polymerase 1) Inhibitory Ability from US Patent US12459899: "Isoquinolinone derivatives, method for preparing the same, and pharmaceutical composition for preventing or treating poly(ADP-ribose) polymerase-1-related diseases, comprising the same as active ingredient"
247	46226354	5.6518	0.0050	5.0000	8.2478	8.3010	455679	20007017	Inhibition of human PARP1 after 3 hrs using [3H]NAD+ by scintillation proximity assay
248	153339051	5.7014	0.0042	4.2000	8.2440	8.3768	1849657	33129590	Displacement of [125I]KX1 from PARP1 in human OVCAR-8 cells incubated for 1 hr by wizard gamma counter analysis
249	156318214	5.7158	0.0010	1.0000	8.2429	9.0000	2202528		PARP-1 (poly[ADP-ribose] polymerase 1) Inhibitory Ability from US Patent US12459899: "Isoquinolinone derivatives, method for preparing the same, and pharmaceutical composition for preventing or treating poly(ADP-ribose) polymerase-1-related diseases, comprising the same as active ingredient"
250	156318211	5.7396	0.0010	1.0000	8.2411	9.0000	2202528		PARP-1 (poly[ADP-ribose] polymerase 1) Inhibitory Ability from US Patent US12459899: "Isoquinolinone derivatives, method for preparing the same, and pharmaceutical composition for preventing or treating poly(ADP-ribose) polymerase-1-related diseases, comprising the same as active ingredient"
251	156317912	5.8094	0.0010	1.0000	8.2359	9.0000	2202528		PARP-1 (poly[ADP-ribose] polymerase 1) Inhibitory Ability from US Patent US12459899: "Isoquinolinone derivatives, method for preparing the same, and pharmaceutical composition for preventing or treating poly(ADP-ribose) polymerase-1-related diseases, comprising the same as active ingredient"
252	136242907	5.8185	0.0049	4.9000	8.2352	8.3098	1683890	33264017	Inhibition of human PARP-1 catalytic domain (662 to 1011 residues) expressed in Escherichia coli BL21(DE3) cells pre-incubated for 30 mins before addition of activated DNA and NAD by fluorescence based assay
253	25133976	5.8240	0.0050	5.0000	8.2348	8.3010	386691; 1798813	18800822	Inhibition of PARP1 by flashplate scintillation proximity assay | PARP-1 Enzyme Assay from Article 10.1021/jm8001263: "4-[3-(4-cyclopropanecarbonylpiperazine-1-carbonyl)-4-fluorobenzyl]-2H-phthalazin-1-one: a novel bioavailable inhibitor of poly(ADP-ribose) polymerase-1."
254	130292410	5.8272	0.0028	2.7900	8.2345	8.5544	1830902	34748333	Inhibition of recombinant human PARP1 using NAD+ as substrate incubated for 1 hr by ELISA
255	145951996	5.8839	0.0071	7.1000	8.2303	8.1487	1499315	28692916	Inhibition of PARP1 (unknown origin) by ELISA
256	137238780	5.9065	0.0018	1.8400	8.2287	8.7352	1276413	26652717	Inhibition of human PARP1 using [3H]NAD as substrate after 1 min by microplate scintillation counting analysis
257	25133282	5.9177	0.0060	6.0000	8.2278	8.2218	386691; 1798813	18800822	Inhibition of PARP1 by flashplate scintillation proximity assay | PARP-1 Enzyme Assay from Article 10.1021/jm8001263: "4-[3-(4-cyclopropanecarbonylpiperazine-1-carbonyl)-4-fluorobenzyl]-2H-phthalazin-1-one: a novel bioavailable inhibitor of poly(ADP-ribose) polymerase-1."
258	164042200	5.9311	0.0049	4.8600	8.2269	8.3134	2019975	37843892	Inhibition of human recombinant PARP1 expressed in Escherichia coli BL21(DE3) incubated for 1 hr by ELISA assay
259	156317951	5.9969	0.0010	1.0000	8.2221	9.0000	2202528		PARP-1 (poly[ADP-ribose] polymerase 1) Inhibitory Ability from US Patent US12459899: "Isoquinolinone derivatives, method for preparing the same, and pharmaceutical composition for preventing or treating poly(ADP-ribose) polymerase-1-related diseases, comprising the same as active ingredient"
260	44407931	6.0006	0.0050	5.0000	8.2218	8.3010	260968	16290932	Inhibitory activity against PARP1

No.	CID	Pred. IC50 (nM)	Exp. IC50 (uM)	Exp. IC50 (nM)	Pred. pIC50	Exp. pIC50	AID(s)	PubMed ID(s)	PARP1 assay name(s)
261	136186322	6.0287	0.0063	6.2900	8.2198	8.2013	1276413	26652717	Inhibition of human PARP1 using [3H]NAD as substrate after 1 min by microplate scintillation counting analysis
262	122707106	6.0409	0.0076	7.5900	8.2189	8.1198	2019975	37843892	Inhibition of human recombinant PARP1 expressed in Escherichia coli BL21(DE3) incubated for 1 hr by ELISA assay
263	145955367	6.0567	0.0705	70.5000	8.2178	7.1518	1499315	28692916	Inhibition of PARP1 (unknown origin) by ELISA
264	156317993	6.0670	0.0010	1.0000	8.2170	9.0000	2202528		PARP-1 (poly[ADP-ribose] polymerase 1) Inhibitory Ability from US Patent US12459899: "Isoquinolinone derivatives, method for preparing the same, and pharmaceutical composition for preventing or treating poly(ADP-ribose) polymerase-1-related diseases, comprising the same as active ingredient"
265	130292391	6.0757	0.0069	6.8800	8.2164	8.1624	1830902	34748333	Inhibition of recombinant human PARP1 using NAD+ as substrate incubated for 1 hr by ELISA
266	156318846	6.1108	0.0010	1.0000	8.2139	9.0000	2202528		PARP-1 (poly[ADP-ribose] polymerase 1) Inhibitory Ability from US Patent US12459899: "Isoquinolinone derivatives, method for preparing the same, and pharmaceutical composition for preventing or treating poly(ADP-ribose) polymerase-1-related diseases, comprising the same as active ingredient"
267	153339069	6.1118	0.0047	4.7000	8.2138	8.3279	1849657	33129590	Displacement of [125I]KX1 from PARP1 in human OVCAR-8 cells incubated for 1 hr by wizard gamma counter analysis
268	156317977	6.1144	0.0010	1.0000	8.2136	9.0000	2202528		PARP-1 (poly[ADP-ribose] polymerase 1) Inhibitory Ability from US Patent US12459899: "Isoquinolinone derivatives, method for preparing the same, and pharmaceutical composition for preventing or treating poly(ADP-ribose) polymerase-1-related diseases, comprising the same as active ingredient"
269	172452888	6.1600	0.0170	17.0500	8.2104	7.7683	2019975	37843892	Inhibition of human recombinant PARP1 expressed in Escherichia coli BL21(DE3) incubated for 1 hr by ELISA assay
270	156317992	6.1990	0.0010	1.0000	8.2077	9.0000	2202528		PARP-1 (poly[ADP-ribose] polymerase 1) Inhibitory Ability from US Patent US12459899: "Isoquinolinone derivatives, method for preparing the same, and pharmaceutical composition for preventing or treating poly(ADP-ribose) polymerase-1-related diseases, comprising the same as active ingredient"
271	46226422	6.2035	0.0060	6.0000	8.2074	8.2218	455679	20007017	Inhibition of human PARP1 after 3 hrs using [3H]NAD+ by scintillation proximity assay
272	156318070	6.2188	0.0010	1.0000	8.2063	9.0000	2202528		PARP-1 (poly[ADP-ribose] polymerase 1) Inhibitory Ability from US Patent US12459899: "Isoquinolinone derivatives, method for preparing the same, and pharmaceutical composition for preventing or treating poly(ADP-ribose) polymerase-1-related diseases, comprising the same as active ingredient"
273	162666779	6.2197	0.0098	9.8000	8.2062	8.0088	1683890	33264017	Inhibition of human PARP-1 catalytic domain (662 to 1011 residues) expressed in Escherichia coli BL21(DE3) cells pre-incubated for 30 mins before addition of activated DNA and NAD by fluorescence based assay
274	71626464	6.2510	0.0066	6.6000	8.2040	8.1805	1683890	33264017	Inhibition of human PARP-1 catalytic domain (662 to 1011 residues) expressed in Escherichia coli BL21(DE3) cells pre-incubated for 30 mins before addition of activated DNA and NAD by fluorescence based assay
275	172439339	6.2583	0.0042	4.1800	8.2035	8.3788	2019975	37843892	Inhibition of human recombinant PARP1 expressed in Escherichia coli BL21(DE3) incubated for 1 hr by ELISA assay
276	156318169	6.2822	0.0010	1.0000	8.2019	9.0000	2202528		PARP-1 (poly[ADP-ribose] polymerase 1) Inhibitory Ability from US Patent US12459899: "Isoquinolinone derivatives, method for preparing the same, and pharmaceutical composition for preventing or treating poly(ADP-ribose) polymerase-1-related diseases, comprising the same as active ingredient"
277	156318274	6.2822	0.0010	1.0000	8.2019	9.0000	2202528		PARP-1 (poly[ADP-ribose] polymerase 1) Inhibitory Ability from US Patent US12459899: "Isoquinolinone derivatives, method for preparing the same, and pharmaceutical composition for preventing or treating poly(ADP-ribose) polymerase-1-related diseases, comprising the same as active ingredient"
278	172459694	6.2902	0.0089	8.8800	8.2013	8.0516	2019975	37843892	Inhibition of human recombinant PARP1 expressed in Escherichia coli BL21(DE3) incubated for 1 hr by ELISA assay
279	46226063	6.3110	0.0060	6.0000	8.1999	8.2218	455679	20007017	Inhibition of human PARP1 after 3 hrs using [3H]NAD+ by scintillation proximity assay
280	172467653	6.3115	0.0051	5.0600	8.1999	8.2958	2019975	37843892	Inhibition of human recombinant PARP1 expressed in Escherichia coli BL21(DE3) incubated for 1 hr by ELISA assay
281	136265176	6.3133	0.0019	1.9500	8.1997	8.7100	1276413	26652717	Inhibition of human PARP1 using [3H]NAD as substrate after 1 min by microplate scintillation counting analysis
282	136186309	6.3204	0.0062	6.1800	8.1993	8.2090	1276413	26652717	Inhibition of human PARP1 using [3H]NAD as substrate after 1 min by microplate scintillation counting analysis

No.	CID	Pred. IC50 (nM)	Exp. IC50 (uM)	Exp. IC50 (nM)	Pred. pIC50	Exp. pIC50	AID(s)	PubMed ID(s)	PARP1 assay name(s)
283	156318111	6.3880	0.0010	1.0000	8.1946	9.0000	2202528		PARP-1 (poly[ADP-ribose] polymerase 1) Inhibitory Ability from US Patent US12459899: "Isoquinolinone derivatives, method for preparing the same, and pharmaceutical composition for preventing or treating poly(ADP-ribose) polymerase-1-related diseases, comprising the same as active ingredient"
284	162524435	6.4072	0.0260	26.0000	8.1933	7.5850	2061152		PARP Fluorescence Anisotropy Binding Assay from US Patent US20250082630: "METHODS OF TREATING BRAIN TUMOURS AND NEUROBLASTOMAS"
285	172463337	6.4139	0.0038	3.7600	8.1929	8.4248	2019975	37843892	Inhibition of human recombinant PARP1 expressed in Escherichia coli BL21(DE3) incubated for 1 hr by ELISA assay
286	25132613	6.4903	0.0100	10.0000	8.1877	8.0000	386691; 1798813	18800822	Inhibition of PARP1 by flashplate scintillation proximity assay | PARP-1 Enzyme Assay from Article 10.1021/jm8001263: "4-[3-(4-cyclopropanecarbonylpiperazine-1-carbonyl)-4-fluorobenzyl]-2H-phthalazin-1-one: a novel bioavailable inhibitor of poly(ADP-ribose) polymerase-1."
287	156319022	6.5268	0.0010	1.0000	8.1853	9.0000	2202528		PARP-1 (poly[ADP-ribose] polymerase 1) Inhibitory Ability from US Patent US12459899: "Isoquinolinone derivatives, method for preparing the same, and pharmaceutical composition for preventing or treating poly(ADP-ribose) polymerase-1-related diseases, comprising the same as active ingredient"
288	118905796	6.5531	0.0033	3.3000	8.1836	8.4815	1261692; 1261693; 1261694	26469301	Inhibition of PARP1 (unknown origin) incubated for 10 mins using biotinylated NAD+ and activated DNA by colorimetric assay | Inhibition of PARP1 in human G7 cells incubated for 60 mins by immunofluorescence assay | Inhibition of PARP1 in human T98G cells incubated for 60 mins by immunofluorescence assay
289	18444338	6.5670	0.0095	9.5000	8.1826	8.0223	241527	15837300	Inhibitory concentration against human poly (ADP-ribose) polymerase 1 (PARP-1)
290	153525072	6.6074	0.0042	4.2300	8.1800	8.3737	1667252	32088129	Inhibition of PARP1 (unknown origin)
291	145960349	6.6614	0.0001	0.1000	8.1764	10.0000	1499315	28692916	Inhibition of PARP1 (unknown origin) by ELISA
292	156318220	6.7045	0.0010	1.0000	8.1736	9.0000	2202528		PARP-1 (poly[ADP-ribose] polymerase 1) Inhibitory Ability from US Patent US12459899: "Isoquinolinone derivatives, method for preparing the same, and pharmaceutical composition for preventing or treating poly(ADP-ribose) polymerase-1-related diseases, comprising the same as active ingredient"
293	136242898	6.7331	0.0052	5.2000	8.1718	8.2840	1683890	33264017	Inhibition of human PARP-1 catalytic domain (662 to 1011 residues) expressed in Escherichia coli BL21(DE3) cells pre-incubated for 30 mins before addition of activated DNA and NAD by fluorescence based assay
294	156318132	6.7672	0.0010	1.0000	8.1696	9.0000	2202528		PARP-1 (poly[ADP-ribose] polymerase 1) Inhibitory Ability from US Patent US12459899: "Isoquinolinone derivatives, method for preparing the same, and pharmaceutical composition for preventing or treating poly(ADP-ribose) polymerase-1-related diseases, comprising the same as active ingredient"
295	156319054	6.7672	0.0010	1.0000	8.1696	9.0000	2202528		PARP-1 (poly[ADP-ribose] polymerase 1) Inhibitory Ability from US Patent US12459899: "Isoquinolinone derivatives, method for preparing the same, and pharmaceutical composition for preventing or treating poly(ADP-ribose) polymerase-1-related diseases, comprising the same as active ingredient"
296	156318006	6.7765	0.0010	1.0000	8.1690	9.0000	2202528		PARP-1 (poly[ADP-ribose] polymerase 1) Inhibitory Ability from US Patent US12459899: "Isoquinolinone derivatives, method for preparing the same, and pharmaceutical composition for preventing or treating poly(ADP-ribose) polymerase-1-related diseases, comprising the same as active ingredient"
297	162649500	6.7894	0.0066	6.6000	8.1682	8.1805	1683890	33264017	Inhibition of human PARP-1 catalytic domain (662 to 1011 residues) expressed in Escherichia coli BL21(DE3) cells pre-incubated for 30 mins before addition of activated DNA and NAD by fluorescence based assay
298	72736758	6.8032	0.0002	0.2000	8.1673	9.6990	1638260; 1638268; 1927623; 1993745; 2138121; 2138123	30684797; 32717529; 38516606; 37567056	Inhibition of recombinant human PARP1 using histone as substrate after 1 hr in presence of NAD+ by ELISA | Inhibition of PARP1 (unknown origin) by NAD+ based assay | Inhibition of PARP1 (unknown origin) | Inhibition of PARP1 (unknown origin) by ELISA | Inhibition of PARP1 (unknown origin) incubated for 1 hr by ELISA analysis
299	76684090	6.8217	0.0054	5.4000	8.1661	8.2676	1638271	30684797	Inhibition of human recombinant PARP1 by chemiluminescence assay
300	25132609	6.8473	0.0080	8.0000	8.1645	8.0969	386691; 1798813	18800822	Inhibition of PARP1 by flashplate scintillation proximity assay | PARP-1 Enzyme Assay from Article 10.1021/jm8001263: "4-[3-(4-cyclopropanecarbonylpiperazine-1-carbonyl)-4-fluorobenzyl]-2H-phthalazin-1-one: a novel bioavailable inhibitor of poly(ADP-ribose) polymerase-1."
301	136242903	6.8754	0.0288	28.8000	8.1627	7.5406	1683890	33264017	Inhibition of human PARP-1 catalytic domain (662 to 1011 residues) expressed in Escherichia coli BL21(DE3) cells pre-incubated for 30 mins before addition of activated DNA and NAD by fluorescence based assay

No.	CID	Pred. IC50 (nM)	Exp. IC50 (uM)	Exp. IC50 (nM)	Pred. pIC50	Exp. pIC50	AID(s)	PubMed ID(s)	PARP1 assay name(s)
302	135924304	6.8842	0.0095	9.4500	8.1621	8.0246	1276413	26652717	Inhibition of human PARP1 using [3H]NAD as substrate after 1 min by microplate scintillation counting analysis
303	156596360	6.8893	0.0050	5.0000	8.1618	8.3010	1895766	34570508	Inhibition of human recombinant N-terminal 6His-6Lys-TEV tagged PARP1 full length expressed in pFastBac expression system incubated for 4 hrs by fluorescence anisotropy binding assay
304	44549647	6.9280	0.0037	3.7000	8.1594	8.4318	477514; 1205269	19873981; 25761096	Inhibition of human PARP1 by SPA | Inhibition of PARP1 (unknown origin)
305	122707105	7.0201	0.0118	11.8100	8.1537	7.9278	2019975	37843892	Inhibition of human recombinant PARP1 expressed in Escherichia coli BL21(DE3) incubated for 1 hr by ELISA assay
306	118136401	7.0207	0.0119	11.9000	8.1536	7.9245	2138123	37567056	Inhibition of PARP1 (unknown origin) incubated for 1 hr by ELISA analysis
307	156318986	7.1046	0.0010	1.0000	8.1485	9.0000	2202528		PARP-1 (poly[ADP-ribose] polymerase 1) Inhibitory Ability from US Patent US12459899: "Isoquinolinone derivatives, method for preparing the same, and pharmaceutical composition for preventing or treating poly(ADP-ribose) polymerase-1-related diseases, comprising the same as active ingredient"
308	168177331	7.1325	0.0034	3.4000	8.1468	8.4685	1896630; 1896632	36343904	Inhibition of PARP-1 (unknown origin) | Inhibition of PARP-1 (unknown origin) binding to DNA assessed as DNA trapping activity
309	25133979	7.1863	0.0150	15.0000	8.1435	7.8239	386691; 1798813	18800822	Inhibition of PARP1 by flashplate scintillation proximity assay | PARP-1 Enzyme Assay from Article 10.1021/jm8001263: "4-[3-(4-cyclopropanecarbonylpiperazine-1-carbonyl)-4-fluorobenzyl]-2H-phthalazin-1-one: a novel bioavailable inhibitor of poly(ADP-ribose) polymerase-1."
310	132576436	7.1887	0.4670	467.0000	8.1433	6.3307	1486467	28622906	Inhibition of recombinant human PARP-1 expressed in Escherichia coli BL21 (DE3) using sheared DNA as substrate in presence of biotinylated NAD after 1 hr by ELISA
311	156318011	7.2091	0.0010	1.0000	8.1421	9.0000	2202528		PARP-1 (poly[ADP-ribose] polymerase 1) Inhibitory Ability from US Patent US12459899: "Isoquinolinone derivatives, method for preparing the same, and pharmaceutical composition for preventing or treating poly(ADP-ribose) polymerase-1-related diseases, comprising the same as active ingredient"
312	25218484	7.2276	0.0050	5.0000	8.1410	8.3010	339995; 1798823	18579376	Inhibition of PARP1 | PARP-1 Enzyme Assay from Article 10.1016/j.bmcl.2008.06.025: "Novel alkoxybenzamide inhibitors of poly(ADP-ribose) polymerase."
313	172444450	7.2320	0.0098	9.8500	8.1407	8.0066	2019975	37843892	Inhibition of human recombinant PARP1 expressed in Escherichia coli BL21(DE3) incubated for 1 hr by ELISA assay
314	138668243	7.2804	0.0058	5.8000	8.1378	8.2366	1729531; 1872256	33309164; 35051747	Inhibition of PARP1 (unknown origin) | Inhibition of PARP1 (unknown origin) incubated for 1 hr by chemiluminescence assay
315	44408056	7.2918	0.0080	8.0000	8.1372	8.0969	260968	16290932	Inhibitory activity against PARP1
316	25133975	7.3060	0.0060	6.0000	8.1363	8.2218	386691; 1798813	18800822	Inhibition of PARP1 by flashplate scintillation proximity assay | PARP-1 Enzyme Assay from Article 10.1021/jm8001263: "4-[3-(4-cyclopropanecarbonylpiperazine-1-carbonyl)-4-fluorobenzyl]-2H-phthalazin-1-one: a novel bioavailable inhibitor of poly(ADP-ribose) polymerase-1."
317	156317964	7.3236	0.0002	0.2000	8.1353	9.6990	2202528		PARP-1 (poly[ADP-ribose] polymerase 1) Inhibitory Ability from US Patent US12459899: "Isoquinolinone derivatives, method for preparing the same, and pharmaceutical composition for preventing or treating poly(ADP-ribose) polymerase-1-related diseases, comprising the same as active ingredient"
318	156318320	7.3335	0.0010	1.0000	8.1347	9.0000	2202528		PARP-1 (poly[ADP-ribose] polymerase 1) Inhibitory Ability from US Patent US12459899: "Isoquinolinone derivatives, method for preparing the same, and pharmaceutical composition for preventing or treating poly(ADP-ribose) polymerase-1-related diseases, comprising the same as active ingredient"
319	44549780	7.3361	0.0037	3.7000	8.1345	8.4318	477514	19873981	Inhibition of human PARP1 by SPA
320	166065636	7.3412	0.0120	12.0000	8.1342	7.9208	1896630; 1896632	36343904	Inhibition of PARP-1 (unknown origin) | Inhibition of PARP-1 (unknown origin) binding to DNA assessed as DNA trapping activity
321	155569079	7.3920	0.0049	4.8980	8.1312	8.3100	1581911	31846325	Inhibition of recombinant human PARP1 using histone as substrate after 1 hr in presence of biotinylated NAD+ by ELISA
322	136242905	7.4008	0.0091	9.1000	8.1307	8.0410	1683890	33264017	Inhibition of human PARP-1 catalytic domain (662 to 1011 residues) expressed in Escherichia coli BL21(DE3) cells pre-incubated for 30 mins before addition of activated DNA and NAD by fluorescence based assay

No.	CID	Pred. IC50 (nM)	Exp. IC50 (uM)	Exp. IC50 (nM)	Pred. pIC50	Exp. pIC50	AID(s)	PubMed ID(s)	PARP1 assay name(s)
323	171347885	7.4780	0.0020	2.0000	8.1262	8.6990	1798813	18800822	PARP-1 Enzyme Assay from Article 10.1021/jm8001263: "4-[3-(4-cyclopropanecarbonylpiperazine-1-carbonyl)-4-fluorobenzyl]-2H-phthalazin-1-one: a novel bioavailable inhibitor of poly(ADP-ribose) polymerase-1."
324	11560961	8.5470	0.0068	6.8000	8.0682	8.1675	241527; 367823	15837300; 19125579	Inhibitory concentration against human poly (ADP-ribose) polymerase 1 (PARP-1) | Inhibition of human recombinant PARP1
325	156318310	8.5471	0.0006	0.6500	8.0682	9.1871	2202528		PARP-1 (poly[ADP-ribose] polymerase 1) Inhibitory Ability from US Patent US12459899: "Isoquinolinone derivatives, method for preparing the same, and pharmaceutical composition for preventing or treating poly(ADP-ribose) polymerase-1-related diseases, comprising the same as active ingredient"
326	156318409	8.5565	0.0010	1.0000	8.0677	9.0000	2202528		PARP-1 (poly[ADP-ribose] polymerase 1) Inhibitory Ability from US Patent US12459899: "Isoquinolinone derivatives, method for preparing the same, and pharmaceutical composition for preventing or treating poly(ADP-ribose) polymerase-1-related diseases, comprising the same as active ingredient"
327	44402241	8.5635	0.0050	5.0000	8.0673	8.3010	241527	15837300	Inhibitory concentration against human poly (ADP-ribose) polymerase 1 (PARP-1)
328	156318152	8.5650	0.0010	1.0000	8.0673	9.0000	2202528		PARP-1 (poly[ADP-ribose] polymerase 1) Inhibitory Ability from US Patent US12459899: "Isoquinolinone derivatives, method for preparing the same, and pharmaceutical composition for preventing or treating poly(ADP-ribose) polymerase-1-related diseases, comprising the same as active ingredient"
329	46226395	8.6077	0.0080	8.0000	8.0651	8.0969	455679	20007017	Inhibition of human PARP1 after 3 hrs using [3H]NAD+ by scintillation proximity assay
330	136242899	8.6116	0.0058	5.8000	8.0649	8.2366	1683890	33264017	Inhibition of human PARP-1 catalytic domain (662 to 1011 residues) expressed in Escherichia coli BL21(DE3) cells pre-incubated for 30 mins before addition of activated DNA and NAD by fluorescence based assay
331	136242913	8.6386	0.0085	8.5000	8.0636	8.0706	1683890	33264017	Inhibition of human PARP-1 catalytic domain (662 to 1011 residues) expressed in Escherichia coli BL21(DE3) cells pre-incubated for 30 mins before addition of activated DNA and NAD by fluorescence based assay
332	136265177	8.6431	0.0096	9.5900	8.0633	8.0182	1276413	26652717	Inhibition of human PARP1 using [3H]NAD as substrate after 1 min by microplate scintillation counting analysis
333	156318488	8.6960	0.0010	1.0000	8.0607	9.0000	2202528		PARP-1 (poly[ADP-ribose] polymerase 1) Inhibitory Ability from US Patent US12459899: "Isoquinolinone derivatives, method for preparing the same, and pharmaceutical composition for preventing or treating poly(ADP-ribose) polymerase-1-related diseases, comprising the same as active ingredient"
334	172442749	8.7035	0.0030	3.0000	8.0603	8.5229	2025285	38088333	Inhibition of human recombinant PARP-1
335	141420190	8.7056	0.0084	8.4100	8.0602	8.0752	1557957	31401008	Inhibition of human PARP1 expressed in Escherichia coli incubated for 10 mins by colorimetric assay
336	130292366	8.7085	0.0005	0.5100	8.0601	9.2924	1830902	34748333	Inhibition of recombinant human PARP1 using NAD+ as substrate incubated for 1 hr by ELISA
337	138667945	8.7410	0.0073	7.3000	8.0584	8.1367	1729531; 1872256	33309164; 35051747	Inhibition of PARP1 (unknown origin) | Inhibition of PARP1 (unknown origin) incubated for 1 hr by chemiluminescence assay
338	57390504	8.7920	0.0160	16.0000	8.0559	7.7959	638690	22153339	Inhibition of recombinant human PARP1 by in vitro assay
339	44402125	8.7952	0.0098	9.8000	8.0558	8.0088	241527	15837300	Inhibitory concentration against human poly (ADP-ribose) polymerase 1 (PARP-1)
340	118905298	8.8527	0.0058	5.8000	8.0529	8.2366	1261692	26469301	Inhibition of PARP1 (unknown origin) incubated for 10 mins using biotinylated NAD+ and activated DNA by colorimetric assay
341	11291932	8.8551	0.0034	3.4000	8.0528	8.4685	260968; 353463; 386691; 1798813	16290932; 19354255; 18800822	Inhibitory activity against PARP1 | Inhibition of human PARP1 | Inhibition of PARP1 by flashplate scintillation proximity assay | PARP-1 Enzyme Assay from Article 10.1021/jm8001263: "4-[3-(4-cyclopropanecarbonylpiperazine-1-carbonyl)-4-fluorobenzyl]-2H-phthalazin-1-one: a novel bioavailable inhibitor of poly(ADP-ribose) polymerase-1."
342	44549649	8.8854	0.0019	1.9000	8.0513	8.7212	477514	19873981	Inhibition of human PARP1 by SPA
343	71626462	8.9053	0.0055	5.5000	8.0504	8.2596	1683890	33264017	Inhibition of human PARP-1 catalytic domain (662 to 1011 residues) expressed in Escherichia coli BL21(DE3) cells pre-incubated for 30 mins before addition of activated DNA and NAD by fluorescence based assay
344	138667943	8.9127	0.0108	10.8000	8.0500	7.9666	1729531	33309164	Inhibition of PARP1 (unknown origin)
345	168931566	8.9550	0.0015	1.5300	8.0479	8.8153	2061048		PARP1/PARP2 Trapping Assay from US Patent US20250051324: "PARP INHIBITOR, PHARMACEUTICAL COMPOSITION COMPRISING SAME, AND USE THEREOF"

No.	CID	Pred. IC50 (nM)	Exp. IC50 (uM)	Exp. IC50 (nM)	Pred. pIC50	Exp. pIC50	AID(s)	PubMed ID(s)	PARP1 assay name(s)
346	25132610	9.0180	0.0120	12.0000	8.0449	7.9208	386691; 1798813	18800822	Inhibition of PARP1 by flashplate scintillation proximity assay | PARP-1 Enzyme Assay from Article 10.1021/jm8001263: "4-[3-(4-cyclopropanecarbonylpiperazine-1-carbonyl)-4-fluorobenzyl]-2H-phthalazin-1-one: a novel bioavailable inhibitor of poly(ADP-ribose) polymerase-1."
347	25133629	9.0202	0.0120	12.0000	8.0448	7.9208	386691; 1798813	18800822	Inhibition of PARP1 by flashplate scintillation proximity assay | PARP-1 Enzyme Assay from Article 10.1021/jm8001263: "4-[3-(4-cyclopropanecarbonylpiperazine-1-carbonyl)-4-fluorobenzyl]-2H-phthalazin-1-one: a novel bioavailable inhibitor of poly(ADP-ribose) polymerase-1."
348	44549778	9.0365	0.0140	14.0000	8.0440	7.8539	477514	19873981	Inhibition of human PARP1 by SPA
349	156318393	9.0948	0.0010	1.0000	8.0412	9.0000	2202528		PARP-1 (poly[ADP-ribose] polymerase 1) Inhibitory Ability from US Patent US12459899: "Isoquinolinone derivatives, method for preparing the same, and pharmaceutical composition for preventing or treating poly(ADP-ribose) polymerase-1-related diseases, comprising the same as active ingredient"
350	156318288	9.1043	0.0010	1.0000	8.0408	9.0000	2202528		PARP-1 (poly[ADP-ribose] polymerase 1) Inhibitory Ability from US Patent US12459899: "Isoquinolinone derivatives, method for preparing the same, and pharmaceutical composition for preventing or treating poly(ADP-ribose) polymerase-1-related diseases, comprising the same as active ingredient"
351	44549779	9.1054	0.0067	6.7000	8.0407	8.1739	477514	19873981	Inhibition of human PARP1 by SPA
352	46226398	9.1587	0.0250	25.0000	8.0382	7.6021	455679	20007017	Inhibition of human PARP1 after 3 hrs using [3H]NAD+ by scintillation proximity assay
353	57401020	9.1709	0.0100	10.0000	8.0376	8.0000	638690	22153339	Inhibition of recombinant human PARP1 by in vitro assay
354	130292365	9.2148	0.0029	2.9200	8.0355	8.5346	1830902	34748333	Inhibition of recombinant human PARP1 using NAD+ as substrate incubated for 1 hr by ELISA
355	118737587	9.2738	0.0038	3.8000	8.0327	8.4202	1205269	25761096	Inhibition of PARP1 (unknown origin)
356	46226330	9.2983	0.0100	10.0000	8.0316	8.0000	455679	20007017	Inhibition of human PARP1 after 3 hrs using [3H]NAD+ by scintillation proximity assay
357	44407888	9.3175	0.0090	9.0000	8.0307	8.0458	260968	16290932	Inhibitory activity against PARP1
358	25132956	9.3552	0.0080	8.0000	8.0289	8.0969	386691; 1798813	18800822	Inhibition of PARP1 by flashplate scintillation proximity assay | PARP-1 Enzyme Assay from Article 10.1021/jm8001263: "4-[3-(4-cyclopropanecarbonylpiperazine-1-carbonyl)-4-fluorobenzyl]-2H-phthalazin-1-one: a novel bioavailable inhibitor of poly(ADP-ribose) polymerase-1."
359	136242920	9.4155	0.0073	7.3000	8.0262	8.1367	1683890	33264017	Inhibition of human PARP-1 catalytic domain (662 to 1011 residues) expressed in Escherichia coli BL21(DE3) cells pre-incubated for 30 mins before addition of activated DNA and NAD by fluorescence based assay
360	44407942	9.4188	0.0060	6.0000	8.0260	8.2218	260968	16290932	Inhibitory activity against PARP1
361	135512456	9.4599	0.0022	2.2000	8.0241	8.6576	344541; 1798824	18713665	Inhibition of PARP1 in human HeLa cells by fluid scintillation counting using [adenylated-32P]NAD as substrate | PARP-1 Enzyme Inhibition Assay from Article 10.1016/j.bmcl.2008.07.091: "Identification of ring-fused pyrazolo pyridin-2-ones as novel poly(ADP-ribose)polymerase-1 inhibitors."
362	136242891	9.5074	0.0067	6.7000	8.0219	8.1739	1683890	33264017	Inhibition of human PARP-1 catalytic domain (662 to 1011 residues) expressed in Escherichia coli BL21(DE3) cells pre-incubated for 30 mins before addition of activated DNA and NAD by fluorescence based assay
363	25132611	9.6685	0.0120	12.0000	8.0146	7.9208	386691; 1798813	18800822	Inhibition of PARP1 by flashplate scintillation proximity assay | PARP-1 Enzyme Assay from Article 10.1021/jm8001263: "4-[3-(4-cyclopropanecarbonylpiperazine-1-carbonyl)-4-fluorobenzyl]-2H-phthalazin-1-one: a novel bioavailable inhibitor of poly(ADP-ribose) polymerase-1."
364	25133631	9.8277	0.0130	13.0000	8.0075	7.8861	386691; 1798813	18800822	Inhibition of PARP1 by flashplate scintillation proximity assay | PARP-1 Enzyme Assay from Article 10.1021/jm8001263: "4-[3-(4-cyclopropanecarbonylpiperazine-1-carbonyl)-4-fluorobenzyl]-2H-phthalazin-1-one: a novel bioavailable inhibitor of poly(ADP-ribose) polymerase-1."
365	156318029	9.8585	0.0010	1.0000	8.0062	9.0000	2202528		PARP-1 (poly[ADP-ribose] polymerase 1) Inhibitory Ability from US Patent US12459899: "Isoquinolinone derivatives, method for preparing the same, and pharmaceutical composition for preventing or treating poly(ADP-ribose) polymerase-1-related diseases, comprising the same as active ingredient"
366	136242896	9.9391	0.0060	6.0000	8.0027	8.2218	1683890	33264017	Inhibition of human PARP-1 catalytic domain (662 to 1011 residues) expressed in Escherichia coli BL21(DE3) cells pre-incubated for 30 mins before addition of activated DNA and NAD by fluorescence based assay
