# Supplementary material for: Interpretable QSAR and Complementary Docking for PARP1 Inhibitor Prioritization: Reliability Stratification and Near-Domain Screening
Source: Pharmaceuticals (Basel). 2026 Apr 7;19(4):584. doi: 10.3390/ph19040584 (PMC13119234; doi:10.3390/ph19040584)
Supplement: Supplementary file 1 [file pharmaceuticals-19-00584-s001.zip › Figure S1.pdf]

Figure S1

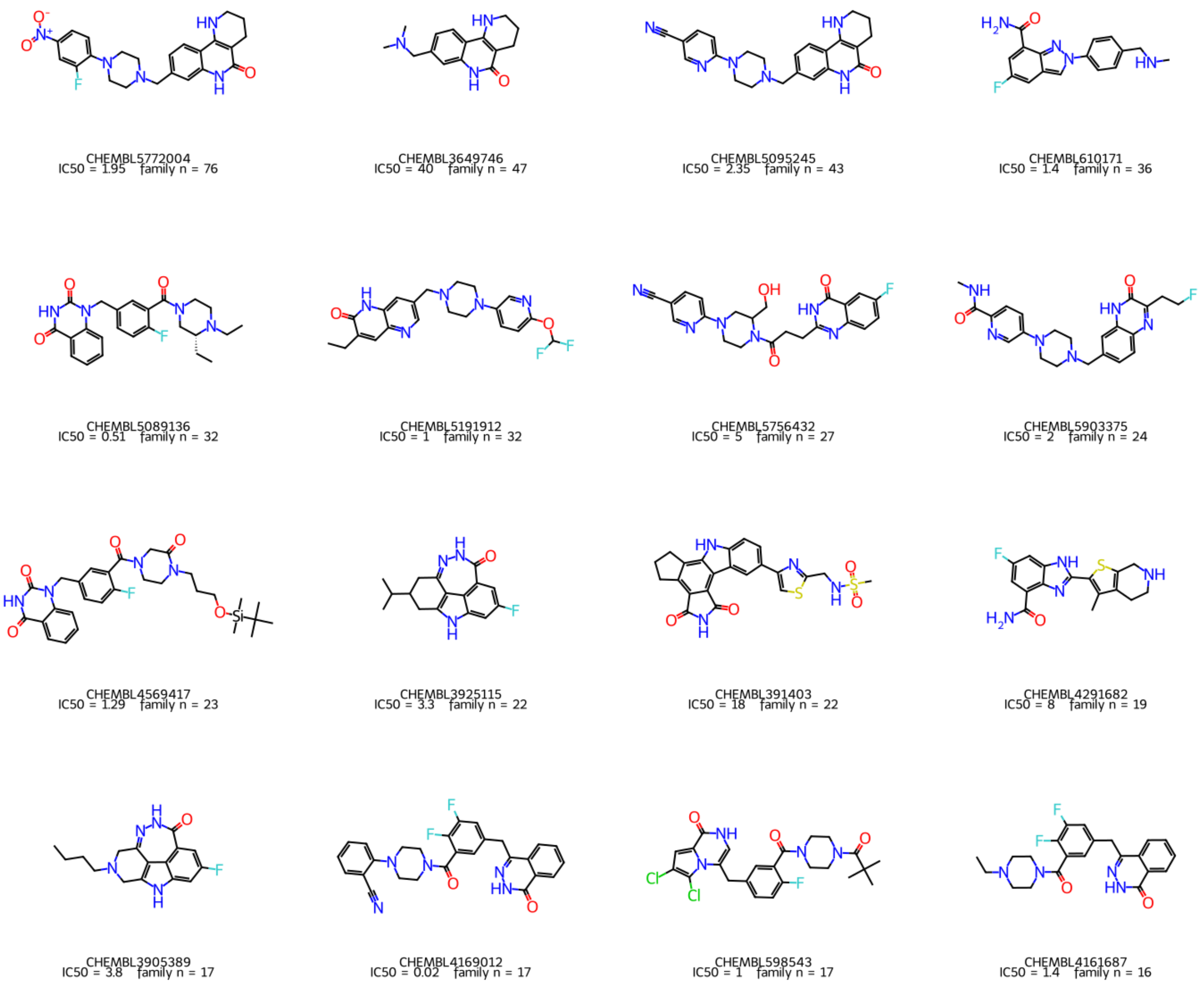

Figure S1. Most frequent Bemis–Murcko scaffold families among PARP1 inhibitors with reported $IC_{50} \leq$ 1000 nM. Representative exemplars are shown for the 16 most populated Bemis–Murcko scaffold families in the active PARP1 inhibitor dataset. Each panel displays one representative compound selected from the corresponding scaffold family, together with its CHEMBL identifier, experimentally reported IC_{50} value, and the total number of molecules assigned to that family. This supplementary figure provides a broader overview of scaffold recurrence and chemical-space organization beyond the eight exemplar ligands shown in Figure 6.
