# Supplementary material for: Interpretable QSAR and Complementary Docking for PARP1 Inhibitor Prioritization: Reliability Stratification and Near-Domain Screening
Source: Pharmaceuticals (Basel). 2026 Apr 7;19(4):584. doi: 10.3390/ph19040584 (PMC13119234; doi:10.3390/ph19040584)
Supplement: Supplementary file 1 [file pharmaceuticals-19-00584-s001.zip › Figure S2.pdf]

Figure S2

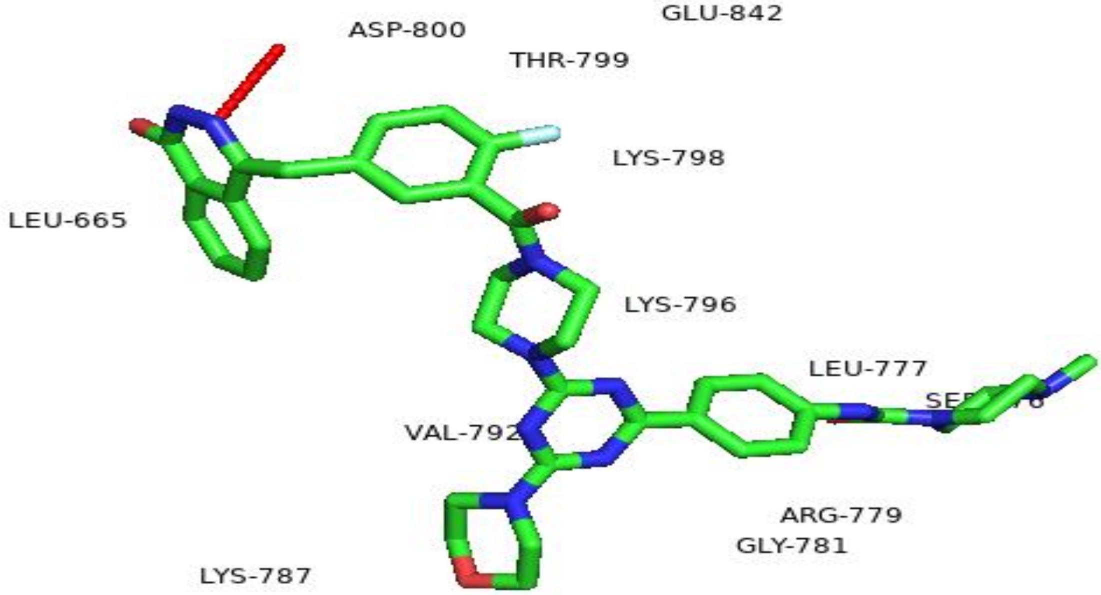

Candidate 6. The ligand is shown in green stick representation and is surrounded by labeled amino acid residues of the binding pocket. A key hydrogen-bond interaction is observed with ASP800 (red dashed lines), while surrounding residues contribute to ligand stabilization through hydrophobic contacts.

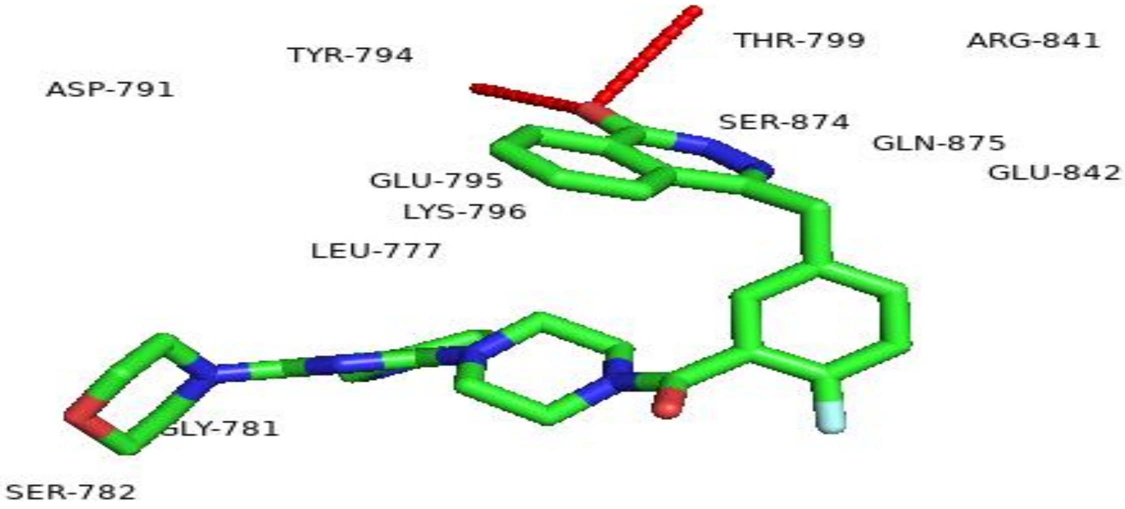

Candidate 5. The ligand is shown in green stick representation within the binding pocket. Key hydrogen-bond interactions are observed with TYR794 and THR799 (red dashed lines), while surrounding residues contribute to stabilization through hydrophobic interactions.

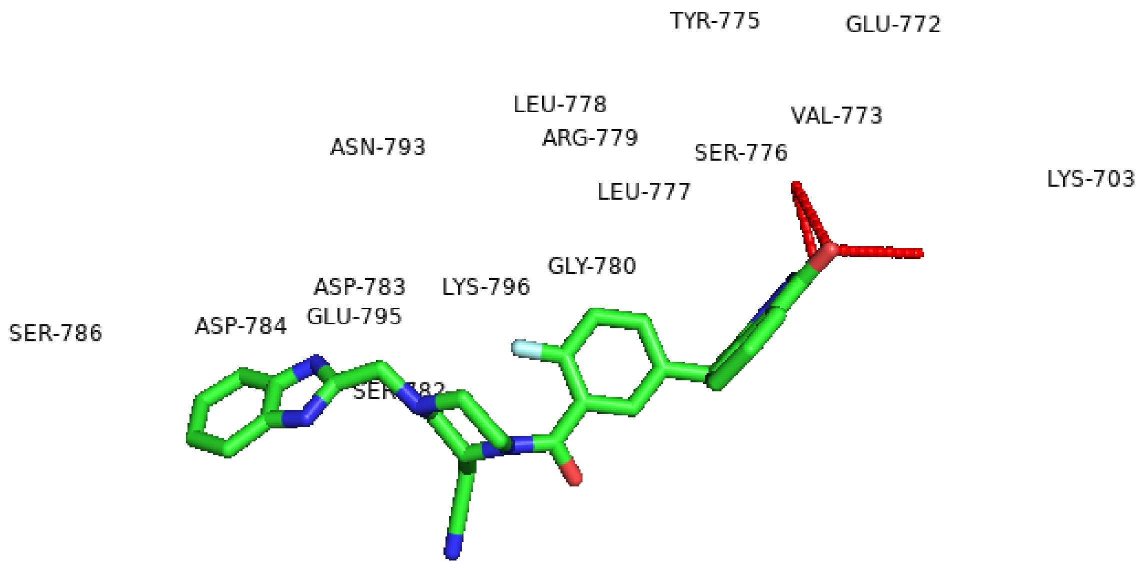

Candidate 9. The ligand is shown in green stick representation within the binding pocket. Key hydrogen-bond interactions are observed with SER776 and LYS703 (red dashed lines), while surrounding residues such as VAL773, LEU777, and GLU795 contribute to ligand stabilization through hydrophobic and polar contacts.

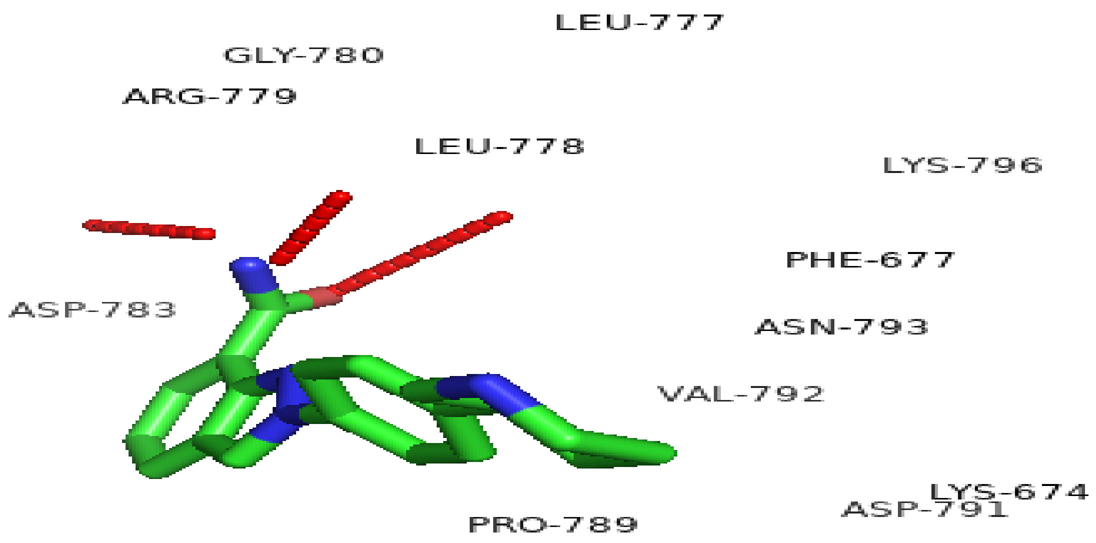

Reference ligand. The ligand is shown in green stick representation within the binding pocket. Hydrogen-bond interactions (red dashed lines) with LYS796, ASP783, and LEU778 contribute to stabilization of the ligand within the active site, while neighboring residues provide additional hydrophobic and electrostatic support.

Figure S2. Stick-style views of the ligand–PARP1 complexes for the three prioritized candidates and the reference ligand. The docked ligands are shown in green stick representation and are surrounded by labeled amino acid residues of the PARP1 binding pocket. Red dashed lines indicate hydrogen-bond interactions. This supplementary figure is provided to complement the cartoon-style representation in Figure 5 by offering a clearer residue-level view of pocket contacts for Candidate 6, Candidate 5, Candidate 9, and the reference ligand (niraparib/3JD)
