# Supplementary material for: Interpretable QSAR and Complementary Docking for PARP1 Inhibitor Prioritization: Reliability Stratification and Near-Domain Screening
Source: Pharmaceuticals (Basel). 2026 Apr 7;19(4):584. doi: 10.3390/ph19040584 (PMC13119234; doi:10.3390/ph19040584)
Supplement: Supplementary file 1 [file pharmaceuticals-19-00584-s001.zip › Supplementary_Tables_S2_S3_S4.pdf]

Supplementary Tables S2–S4

These supplementary tables summarize the final docking shortlist and the comparative AutoDock Vina and Attracting Cavities results used to support structure-based prioritization of QSAR-selected PARP1 candidates.

Supplementary Table S2. Top ten scaffold-diverse PubChem candidates selected for docking against PARP1

The final docking shortlist was selected to balance predicted potency, scaffold-level diversity, and prospective novelty. By applying Bemis–Murcko scaffold filtering after potency ranking, the selection avoided overrepresentation of closely related analog series and increased the likelihood of identifying structurally distinct ligands capable of engaging the PARP1 pocket in chemically plausible ways.

Rank / Candidate	PubChem CID	Predicted IC_{50} (nM)	Selection rationale	SMILES
1	71576509	0.90	Highest-ranked remaining hit without PARP1 assay evidence and with an independent scaffold	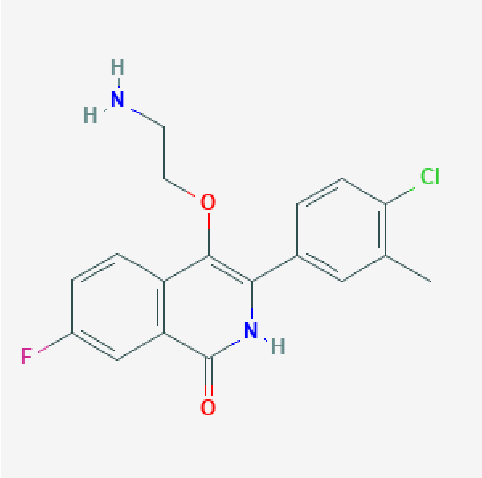
2	175360042	1.08	Highly potent predicted hit with a distinct scaffold	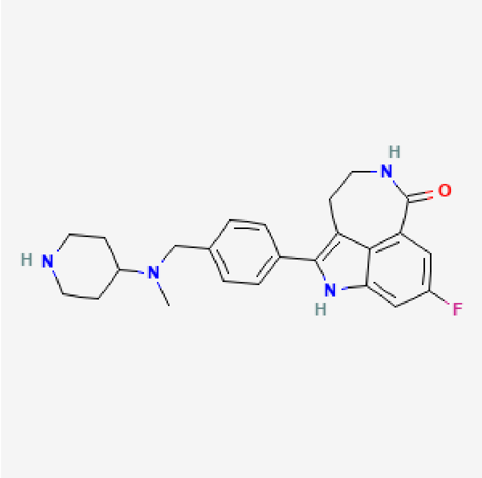

Rank / Candidate	PubChem CID	Predicted IC_{50} (nM)	Selection rationale	SMILES
3	142736906	1.08	Strong candidate with a different chemotype	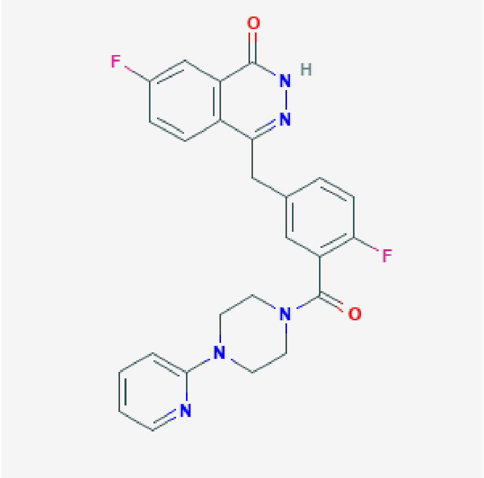
4	22266715	1.11	Structurally distinct chemotype suitable for comparison	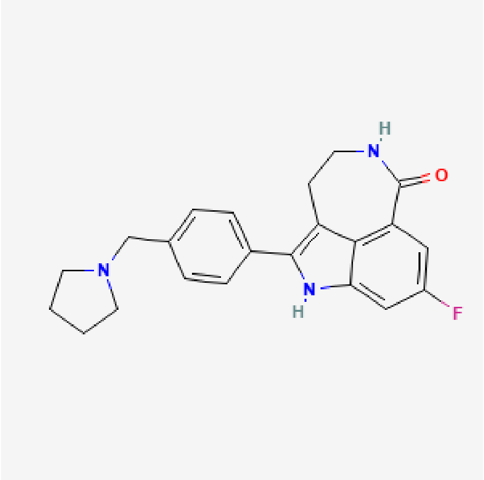

Rank / Candidate	PubChem CID	Predicted IC_{50} (nM)	Selection rationale	SMILES
5	175154210	1.12	Potent hit with an independent scaffold	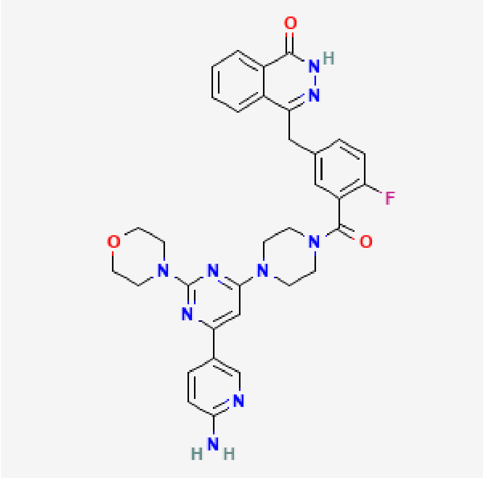
6	168873053	1.13	Unique scaffold and a strong docking candidate	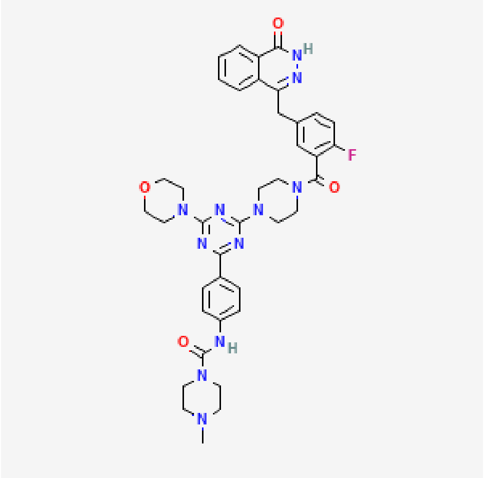

Rank / Candidate	PubChem CID	Predicted IC_{50} (nM)	Selection rationale	SMILES
7	126602454	1.17	Expands chemical-space coverage	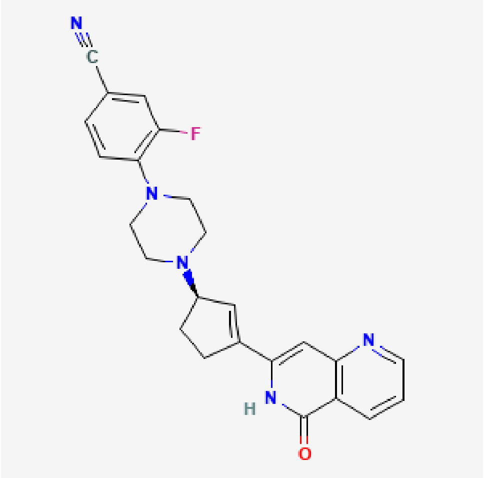
8	150420374	1.24	Structurally different from the higher-ranked clusters	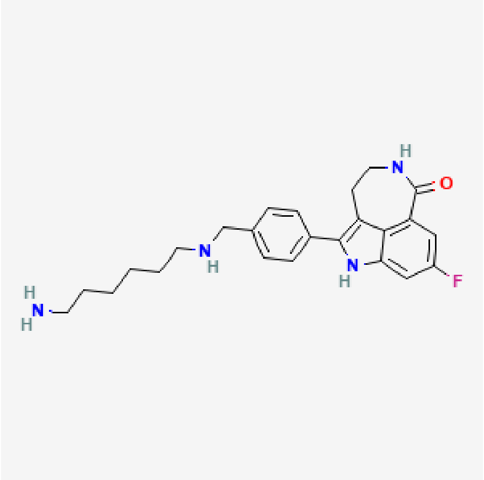

Rank / Candidate	PubChem CID	Predicted IC_{50} (nM)	Selection rationale	SMILES
9	172894737	1.25	Adds another distinct chemotype	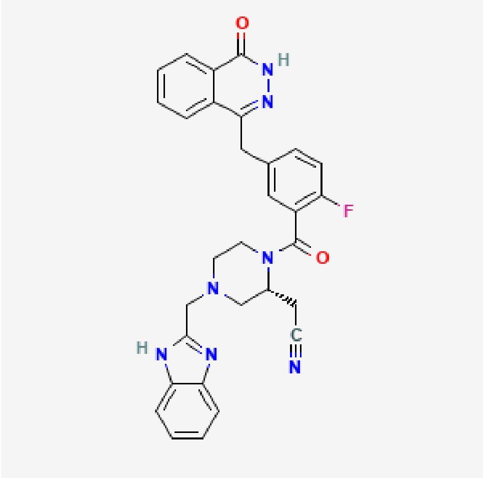
10	168206741	1.27	Maintains diversity with still-strong predicted potency	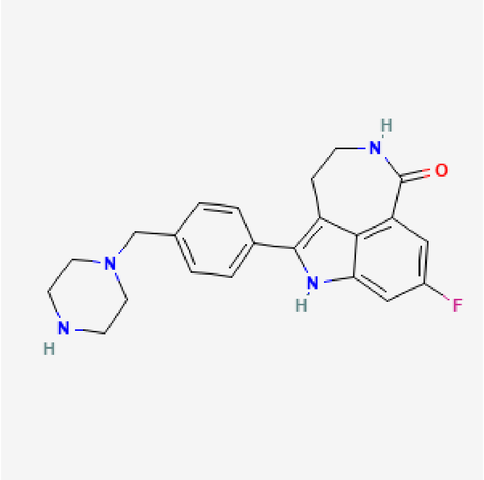

Rank / Candidate	PubChem CID	Predicted IC_{50} (nM)	Selection rationale	SMILES
Ref.	24958200	4.5 (pred.); 3.2 (exp.)	PDB 3JD (4R6E co-crystal ligand), niraparib	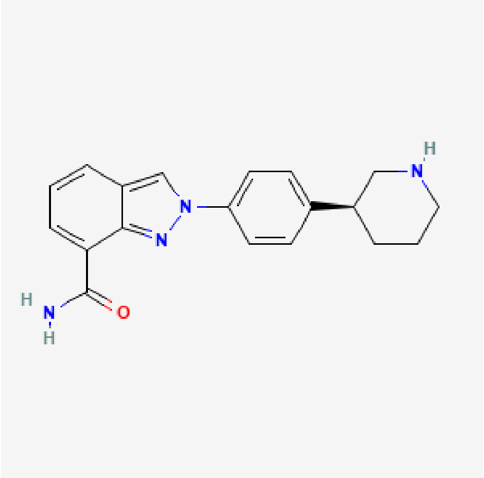

Supplementary Table S3. Comparative summary of AutoDock Vina docking results for Candidates 1–10 and the niraparib reference ligand

Because the SwissDock web server limits individual AutoDock Vina jobs to approximately 10 min, exhaustiveness could not be kept fully identical across all exploratory Vina runs. In each case, search depth was increased as much as permitted within the server runtime limit. Therefore, the Vina scores were treated as provisional and supportive, whereas the fully standardized Attracting Cavities results were used as the primary basis for final docking prioritization.

Ligand	Best Vina score (kcal/mol)	Exhaustiveness	Main nearby residues in top pose	Interpretation / priority
Candidate 1	−5.837	64	PRO789, ASP783, LEU778, VAL792, ASN793, ARG779, SER782, ASP784	Lower-priority candidate; the pose is locally stable, but the score is clearly less favorable than the reference.
Candidate 2	−6.147	30	LEU778, LYS674, LYS796, SER782, ASP783, ARG779, ASP784, ASN793	Lower-priority candidate; pocket-localized pose, but weaker than the reference under the current settings.
Candidate 3	−8.107	30	ILE673, PHE677, ARG779, ASN793, LEU778, ILE790, LYS674, VAL679	High-priority candidate for standardized re-docking; strongest provisional Vina score in the set.
Candidate 4	−5.868	30	LEU778, ASP783, ASN793, SER782, LYS796, PHE677, ARG779, PRO789	Lower-priority candidate; weak score and not competitive with the reference.
Candidate 5	−7.356	10	THR799, LYS798, TYR794, ASP784, GLY781, LEU777, GLU842, GLU795	High-priority candidate for standardized re-docking; favorable score, but obtained with low exhaustiveness.
Candidate 6	−7.486	5	GLU795, ASP800, GLU842, LYS798, SER776, ASP784, LYS796, LEU777	High-priority candidate for standardized re-docking; promising score, but obtained with very low exhaustiveness.
Candidate 7	−6.986	64	GLY781, SER786, LEU777, VAL792, SER782, SER776, LYS796, LYS798	Secondary-priority candidate; standardized run, but score remains slightly weaker than the reference.
Candidate 8	−5.343	20	THR799, TYR794, GLU842, GLU795, LYS798, ASP784, GLY781, SER782	Lower-priority candidate; weakest-scoring ligand in the set.
Candidate 9	−7.509	15	SER776, GLY781, ASP784, GLU795, LEU777, LYS796, GLN875, LYS798	High-priority candidate for standardized re-docking; favorable score, but obtained with non-uniform exhaustiveness.
Candidate 10	−5.965	30	ASP784, ASN793, LYS674, LYS787, ARG779, PHE677, SER782, ASP783	Lower-priority candidate; pocket-localized pose, but not competitive by score.
Reference (niraparib / 3JD)	−7.266	64	VAL679, LEU778, ASP783, GLY780, PHE677, ASP678, LYS796, ARG779	Internal benchmark under the same receptor and site definition.

Interpretation

Comparative AutoDock Vina docking of the ten scaffold-diverse candidates showed that several ligands achieved scores comparable to or more favorable than the niraparib reference. However, because the exploratory Vina runs were performed with non-uniform exhaustiveness settings, the resulting scores should be interpreted cautiously and not used as the sole basis for definitive ranking. Within this provisional screen, Candidates 3, 5, 6, and 9 emerged as the most promising ligands for further evaluation. Candidate 3 showed the most favorable Vina score overall, whereas Candidates 5, 6, and 9 also produced favorable scores but require standardized comparison because their runs used lower search exhaustiveness. Thus, the principal value of Table S3 is to identify pose-compatible, Vina-supported candidates for subsequent comparison against the more standardized cavity-guided docking results.

Supplementary Table S4. Comparative summary of Attracting Cavities docking results for Candidates 1–10 and the reference ligand

Ligand	Best AC SP-dG (kcal/mol)	Main nearby residues in top pose	Recommendation status
Candidate 1	−6.8529	ARG779, PHE677, ASP678, ILE790, VAL679, ILE673, ASN793, LEU778	Comparable to the reference, but not among the strongest AC-supported hits.
Candidate 2	−7.2822	LYS683, ASN793, LYS674, PHE677, ILE790, ASP678, GLU680, VAL679	Moderate AC support; secondary-priority candidate.
Candidate 3	−7.3332	LYS796, ASP784, ASN793, LEU778, ASP678, GLY780, ARG779, VAL792	Strong AC support; remains an important candidate in the shortlist.
Candidate 4	−6.7023	LYS683, PHE677, ASN793, VAL679, GLU680, LYS674	Lower-priority AC result.
Candidate 5	−8.0426	ARG779, PHE677, LYS796, ASP783, ASN793, ASP678, GLY780, LEU778	High-priority consensus candidate.
Candidate 6	−8.5027	LYS796, ARG779, LEU778, LYS674, ASP678, GLY780, VAL679, ILE790	Top AC performer; highest-priority candidate overall in the AC analysis.
Candidate 7	−7.0573	PHE677, ASP678, LYS674, GLU680, ILE790, LYS683, VAL679, ASN793	Moderate AC support.
Candidate 8	−7.1011	PHE677, LYS796, LEU778, GLY780, ASP678, ASP783, VAL792, ARG779	Moderate AC support.
Candidate 9	−7.9488	LYS796, ASP783, ASN793, LEU778, LYS674, ASP678, GLY780, ARG779	High-priority consensus candidate.
Candidate 10	−6.8703	LEU778, ASP783, LYS796, GLY780, LYS674, PRO789, VAL792, ARG779	Comparable to the reference, but not among the strongest AC-supported hits.
Reference (niraparib / 3JD)	−6.8549	LYS683, ASN793, VAL679, GLU680, PHE677, ILE673, ASP678, LYS674	Baseline comparator.

Interpretation

Attracting Cavities docking was performed under fully standardized conditions for all ten scaffold-diverse candidates and the niraparib reference ligand, enabling direct comparison of cavity-guided docking scores. Among the tested compounds, Candidates 6, 5, and 9 showed the most favorable SP-dG values (−8.5027, −8.0426, and −7.9488 kcal/mol, respectively), all exceeding the reference ligand niraparib (−6.8549 kcal/mol). Candidate 3 also showed favorable cavity-guided support (−7.3332 kcal/mol) and remained notable because it was the strongest scorer in the exploratory AutoDock Vina screen. Overall, the Attracting Cavities results supported Candidates 6, 5, and 9 as the most promising ligands for further structural analysis, with Candidate 3 retained as an important cross-method comparator.
